# Impact and feasibility of a group-based therapeutic exercise program on strength and endurance in hospitalized patients with spinal cord injury: a quasi-experimental study

**DOI:** 10.1186/s12984-025-01845-z

**Published:** 2026-02-16

**Authors:** Aitor Garay-Sánchez, Mercedes Ferrando-Margelí, María Navarro-Segura, Aloña Fernández-Celaya, Elena Orejuela-Aparicio, Juan Nicolas Cuenca-Zaldivar, Eleuterio A. Sánchez-Romero, Yolanda Marcén-Román

**Affiliations:** 1 Miguel Servet University Hospital, 50009 Zaragoza, Spain; 2 Institute for Health Research Aragón (IIS Aragón), Zaragoza, Spain; 3 Department of Human Anatomy and Histology, University of Zaragoza, 50009 Zaragoza, Spain; 4 Foundation for Research and Biomedical Innovation in Primary Care (FIIBAP), 28003 Madrid, Spain; 5 Research Group in Nursing and Health Care, Puerta de Hierro Health Research Institute Segovia de Arana (IDIPHISA), Madrid, Spain; 6 Interdisciplinary Research Group on Musculoskeletal Disorders, 28014 Madrid, Spain; 7 Primary Health Center Care El Abajón, Las Rozas de Madrid, Madrid, Spain; 8 Department of Rehabilitation Sciences, Florida Gulf Coast University, Fort Myers, FL USA; 9 Physiotherapy and Orofacial Pain Working Group, Sociedad Española de Disfunción Craneomandibular y Dolor Orofacial (SEDCYDO), Madrid, Spain

**Keywords:** Spinal cord injury, Rehabilitation, Therapeutic exercise, Feasibility, Group training, 6-minute walk, Strength training

## Abstract

**Background:**

Spinal cord injury (SCI) is associated with significant impairment in mobility, muscular strength, and aerobic capacity. Inpatient rehabilitation protocols may not consistently include structured group-based physical training. The structured group-based therapeutic exercise program was designed to address this gap through a supervised therapeutic exercise circuit adapted to the patient’s functional status.

**Objective:**

To evaluate the effectiveness and feasibility of a 12-week group-based therapeutic exercise program on muscular strength, aerobic capacity, adherence, and satisfaction in hospitalized patients with spinal cord injury (SCI).

**Methods:**

A quasi-experimental, single-group pre–post study was conducted in the Neurorehabilitation Unit of Miguel Servet University Hospital (Zaragoza, Spain). Fifty-seven adults with SCI below C3 level were included. Both complete and incomplete cases (AIS A–D) were considered eligible for inclusion. Patients were grouped based on their trunk control ability (seated or standing). The intervention included aerobic and strength training sessions (five days/week). Outcome measures included muscular strength (Medical Research Council Scale), aerobic capacity [6-Minute Walk Test (6MWT) for ambulatory participants or 6-Minute Push Test (6MPT) for wheelchair users], and feasibility (adherence/attendance, patient satisfaction, and safety). Statistical analysis was performed using regression models informed by a directed acyclic graph (DAG), including linear and generalized additive models with permutation-based robustness checks.

**Results:**

Post-intervention improvements were observed in upper-limb strength (mean difference + 3.7 points, 95% CI 2.5–4.9; Cohen’s *d* = 0.62) and 6MWT/6MPT distance (mean difference: +87.5 m, 95% CI 68–108; Cohen’s *d* = 0.55). The effects were more pronounced in participants with AIS C injuries of infectious–vascular origin and in ensure proper sentence structure. Most pre-treatment values did not show a direct association with post-intervention outcomes, suggesting minimal confounding factors. Median attendance was high (39/60 planned sessions; IQR values), satisfaction scores were high overall, no adverse events occurred, and equipment adequacy ratings were lower in non-ambulatory participants.

**Conclusions:**

A supervised, stratified group-based program was feasible and acceptable during inpatient SCI rehabilitation and associated with improvements in muscular strength and aerobic capacity. Given the single-group design and concurrent routine care, these findings were preliminary. Multicenter randomized trials, stratified by AIS and time since injury and incorporating pre-specified feasibility endpoints (safety, adherence, therapist time, satisfaction, and resource use), are warranted to confirm efficacy and evaluate durability and cost-effectiveness.

**Supplementary Information:**

The online version contains supplementary material available at 10.1186/s12984-025-01845-z.

## Introduction

 Spinal cord injury (SCI) is a life-altering condition that profoundly affects an individual’s physical, psychological, and social well-being, with a high global prevalence and substantial impact on health systems. Therefore, effective rehabilitation strategies are essential for enhancing functional recovery and quality of life in this population [1]. In recent years, structured exercise programs have emerged as a cornerstone of neurorehabilitation because they promote neuroplasticity, improve motor function, and mitigate secondary health complications commonly associated with SCI [2]. These interventions aimed to restore mobility, preserve independence, and reduce the long-term burden of disability.

Among the different delivery models, community-based and group-oriented exercise programs have demonstrated promising outcomes. Community-based interventions, for example, facilitate physical activity after discharge and are associated with improvements in cardiovascular health and muscular strength while also fostering social engagement and adherence, two critical factors for long-term rehabilitation success [3, 4]. Within the inpatient setting, group-based therapeutic exercises have gained attention. By providing a structured environment with peer interaction and shared motivation, group formats can reduce pain and disability, enhance adherence, and improve overall well-being in patients with chronic conditions including SCI [5, 6].

Simultaneously, innovative exercise modalities have broadened rehabilitation landscapes. Locomotor training, which emphasizes repetitive task-specific movements, has shown potential to improve walking ability and functional independence by stimulating neural pathways and motor learning [7, 8]. Teleexercise interventions, often using synchronous delivery and focusing on aerobic and strength training, have also demonstrated feasibility, supporting exercise adherence and positive health outcomes in people with SCI [9]. Furthermore, evidence-based scientific exercise guidelines highlight that structured training can significantly improve cardiorespiratory fitness, cardiometabolic health, and muscular strength, underscoring the importance of adherence to standardized protocols in maximizing rehabilitation outcomes [10].

To ensure rigor in evaluating such interventions, nonrandomized studies should be reported transparently using frameworks such as the TREND (Transparent Reporting of Evaluations with Nonrandomized Designs) statement, which facilitates replication and critical appraisal [11]. Despite the growing body of literature, there remains a lack of studies that specifically assess structured group-based exercise programs during inpatient rehabilitation for SCI. Addressing this gap is crucial for determining their effectiveness and feasibility in real-world hospital contexts [12].

This study aimed to evaluate the impact and feasibility of a structured 12-week group-based therapeutic exercise program on muscular strength and aerobic endurance in hospitalized patients with SCI. Feasibility was assessed not only through adherence and patient satisfaction but also through safety and implementation within the inpatient rehabilitation workflow.

## Methods

### Study design and participants

This study was designed as a single-group, prospective, quasi-experimental pre-post intervention trial to assess the effects of a structured group-based therapeutic exercise program (EINTER^®^) on hospitalized patients with spinal cord injury (SCI). The study design was selected owing to ethical and organizational constraints that precluded the random allocation or inclusion of a comparison group.

The intervention was conducted at the Neurorehabilitation Unit (U.L.M.E.) of Miguel Servet University Hospital in Zaragoza, Spain, between January and August 2024. The study protocol was registered at ClinicalTrials.gov (NCT06624566) and approved by the Research Ethics Committee of the Autonomous Community of Aragón (Approval ID: CI PI23/526).

This manuscript was prepared in accordance with the *TREND (Transparent Reporting of Evaluations with Nonrandomized Designs)* guidelines to ensure transparency, replicability, and high-quality reporting of nonrandomized evaluations [11].

### Participants

Eligible participants were adult patients (≥ 18 years) with a confirmed clinical diagnosis of spinal cord injury below the C3 neurological level, who were admitted for inpatient rehabilitation to the Neurorehabilitation Unit (U.L.M.E.) of Miguel Servet University Hospital in Zaragoza, Spain. Patients were consecutively screened for eligibility between January 1 and August 31, 2024.

To reduce heterogeneity, the patients were stratified into functional groups according to trunk control (seated vs. standing). Both complete and incomplete SCI cases were included, as classified using the American Spinal Injury Association Impairment Scale (AIS A–D). The exclusion criteria were tetraplegia at the C3 level or higher, severe medical instability precluding participation in a therapeutic exercise program, severe orthostatic hypotension, active pressure ulcers, deep vein thrombosis, and inability to provide informed consent.

Prior to enrollment, the patients were informed of the study procedures and voluntarily signed a written informed consent form in accordance with the Declaration of Helsinki. Recruitment was conducted in collaboration with the attending clinical rehabilitation teams.

### Intervention

**Fig. 1:**
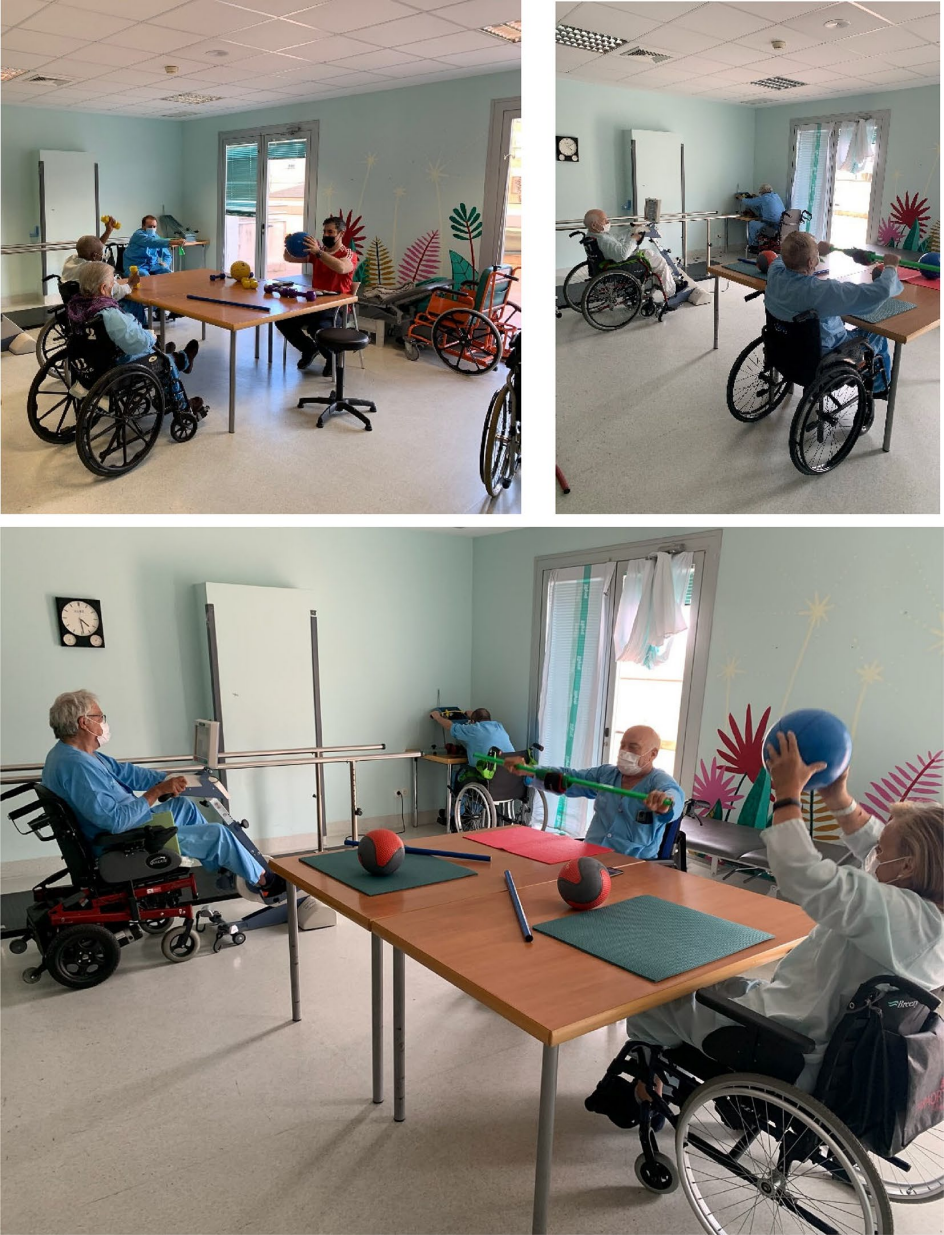
Representative images of the group-based therapeutic exercise program (EINTER^®^) conducted in the inpatient neurorehabilitation unit.Participants with different functional profiles performed supervised aerobic and strength exercises in a structured circuit format, using adapted equipment and stratified according to trunk control, under a 1:4 physiotherapist-to-patient ratio

The intervention consisted of a structured, group-based therapeutic exercise program named EINTER^®^, delivered five days per week (Monday to Friday) over a 12-week period, with each session lasting approximately 45 min. All sessions were conducted in the multifunctional therapy room of the Neurorehabilitation Unit at Miguel Servet University Hospital. Representative images of the group-based therapeutic exercise sessions, illustrating the functional stratification, exercise modalities, and inpatient clinical setting, are shown in Fig. 1.

The participants were assigned to one of two functional groups based on their ability to maintain trunk control, as assessed using the Berg Balance Scale:

- *Group A* Seated trunk control for ≥ 2 min without independent standing.
- *Group B* Standing trunk control for ≥ 2 min.

Patients who were unable to maintain trunk control for at least 2 min in the seated position were excluded for safety reasons. During the recruitment period, no patient was excluded based solely on this criterion.

To ensure safety, each group was continuously supervised by a physiotherapist specializing in neurological rehabilitation (maximum ratio, 1:4). Vital signs were checked before and after each session, and patients were closely monitored during training. Predefined cessation criteria included signs of autonomic instability (e.g., dizziness, hypotension, and autonomic dysreflexia) or musculoskeletal discomfort. All sessions followed institutional safety protocols, and no adverse events occurred.

Each group included a maximum of four participants and was supervised by a single physiotherapist specializing in neurological rehabilitation. The weekly training program was standardized as follows:

- Monday and Wednesday: aerobic training: stationary bicycle (Group B) or motorized exercise device (MOTOmed; Group A). Each session included a 5 min warm-up (joint mobility and stretching), 30 min aerobic effort at 70–90% of the estimated maximum heart rate (220 − age), and 10 min cool-down.
- Tuesday and Thursday – Strength training: Circuit targeting large muscle groups (hip extensors/flexors, abductors/adductors, knee flexors/extensors, and ankle dorsiflexors/plantar flexors). Exercises combined concentric and eccentric actions using free weights, resistance bands, and medicine balls. The strength circuit included both multi-joint and isolated exercises (e.g., seated leg press, knee extensions with elastic bands, hip abduction/adduction, biceps curls, shoulder presses, and medicine ball chest passes). Training loads were set at approximately 50–70% of the estimated one-repetition maximum (1-RM), with 2–3 sets of 10–15 repetitions per exercise adjusted to the patient’s tolerance and safety.
- Friday – Combined training: 20 min aerobic + 15 min strength + 10 min cool-down. Balance and trunk stability exercises were integrated and tailored to each functional group to promote postural control and safety.

The intervention was integrated into each patient’s rehabilitation schedule and did not replace the standard physiotherapy care. Adherence was monitored using attendance logs maintained by the physiotherapists. The program was delivered in parallel with the conventional individualized physiotherapy sessions prescribed by the rehabilitation team, ensuring the continuity of standard care. Exercise intensity and resistance progressed individually according to patient tolerance and clinical response within the predefined aerobic and strength-training framework.

A structured weekly schedule was implemented, as summarized in Table 1.

**Table 1:** Structured group-based therapeutic exercise program (EINTER^®^). Weekly session schedule, type of exercise, examples, intensity, and progression

Day	Session type	Main activities	Intensity / Load	Repetitions / Duration	Progression
Monday	Aerobic	Stationary bicycle (Group B); motorized cycle ergometer – MOTOmed (Group A); warm-up (joint mobility, stretching); cool-down	70–90% of age-predicted max HR (220–age)	5 min warm-up, 30 min aerobic effort, 10 min cool-down	Gradual increase in resistance or speed every 2 weeks if tolerated
Tuesday	Strength	Circuit training for large muscle groups (hip extensors/flexors, abductors/adductors, knee flexors/extensors, ankle dorsiflexors/plantar flexors) using free weights, elastic bands, medicine balls	50–70% of estimated 1RM	2–3 sets × 10–15 reps	Progressive overload: ↑ resistance (band tension, weight) every 2–3 weeks
Wednesday	Aerobic	Same as Monday	Same as Monday	Same as Monday	Same as Monday
Thursday	Strength	Same as Tuesday	Same as Tuesday	Same as Tuesday	Same as Tuesday
Friday	Combined	Aerobic (cycle ergometer or MOTOmed) + strength circuit (large muscle groups) + cool-down	Aerobic: 70–90% HRmax; Strength: 50–70% 1RM	20 min aerobic + 15 min strength + 10 min cool-down	Integrated progression as above

Abbreviations: HRmax, maximal heart rate; 1RM, one-repetition maximum. Progression was individualized based on tolerance and safety with continuous monitoring by a physiotherapist (1:4 ratio)

### Outcome measures

The primary outcome measures were muscular strength and aerobic endurance, which were evaluated before and after the 12-week intervention.

- Muscular strength was assessed using the Medical Research Council (MRC) scale, a standardized 6-point ordinal scale widely used to evaluate voluntary muscle contraction in clinical populations. The MRC scale was chosen over the ISNCSCI motor score because it provides a rapid and clinically feasible assessment applicable to both ambulatory and non-ambulatory patients during routine rehabilitation sessions. While ISNCSCI remains the gold standard for neurological classification, the MRC scale is validated, highly correlated with ISNCSCI motor scores, and has frequently been used in SCI rehabilitation studies [13]. Importantly, the MRC scale also demonstrates high inter-rater reliability in patients with chronic incomplete SCI (Dupépé et al. 2019), supporting its use in clinical and research settings [14]. In this study, the key lower-limb muscle groups (hip, knee, and ankle) were scored bilaterally by experienced physiotherapists.
- Aerobic endurance was evaluated using the 6-Minute Walk Test (6MWT) for ambulatory participants and 6-Minute Push Test (6MPT) for wheelchair users. Both tests followed international protocols and were performed under standardized conditions, with rest periods and monitoring of vital signs. These tests have been validated in individuals with SCI and are responsive to changes.

Secondary outcome measures included:

- Adherence was defined as the number of sessions attended, out of the total schedule. Adherence was monitored using the attendance logs.
- Patient satisfaction at hospital discharge was assessed using a structured satisfaction survey that included Likert-type items on perceived benefit, enjoyment, and willingness to continue exercising after discharge.

All assessments were performed by blinded physiotherapists, who were not involved in the delivery of the intervention. Outcome data were collected at baseline and again within 72 h of discharge from the rehabilitation unit. Intermediate assessments (after 20, 40, or 60 sessions) were performed for descriptive purposes.

### Statistical analysis

Statistical analyses were performed using R (v4.1.3; R Foundation for Statistical Computing, Vienna, Austria). The significance level was set at *P* < 0.05.

Quantitative variables are described as mean ± standard deviation, and qualitative variables are described as absolute and relative values (%).

The sample size was calculated using the study itself as an internal pilot study, with the first 25 patients recruited for a regression model between the average total strength in the upper limbs post-treatment as the dependent variable and post-treatment modulated by the number of sessions received as predictor variables.

A Directed Acyclic Graph (DAG) based on a literature review was used to determine the minimum set of variables to be adjusted to estimate both the direct effect (adjusting for both moderating and mediating variables) and total effect (adjusting only for moderating variables) of pre-treatment muscular strength and functional capacity on post-treatment values [15].

A regression model was applied between the predictor variables and each of the dependent variables of muscular strength and functional capacity after evaluating the presence of multicollinearity using the Pearson or polyserial correlation matrix depending on the type of variable (quantitative or qualitative), eliminating those with a correlation greater than 0.7 (Supplementary material. Table 1). The assumption of linearity between the dependent and predictor variables was tested by taking the value of the effective degrees of freedom (EDF) greater than 2 as the cut-off point (Supplementary material. Table 2). If all variables met this assumption, a linear regression model (LM) was applied; otherwise, a generalized additive model (GAM) was applied with the double-penalty method in the selection of variables. In both cases, compliance with the residual assumptions was checked using the Kolmogorov-Smirnov test with Lilliefors correction (normality of residuals), the Breusch–Pagan test (heteroskedasticity), and the Durbin-Watson test (autocorrelation of residuals). In the GAM models, compliance with the concurvity assumption was also tested for the smoothed terms, eliminating those with a value greater than 0.8 (Supplementary material. Table 3). The adequacy of the number of basic functions was tested using the K-index (*p* > 0.05 as a selection criterion). Because not all residual assumptions were met in all the models, robust versions of the models were used using permutation tests. When model assumptions were not met, permutation-based robust alternatives were applied, thereby ensuring valid inferences under both parametric and non-parametric conditions.

In the ad hoc satisfaction survey, the presence of significant differences based on functional status was tested using the Mann-Whitney U test.

In addition to the prespecified outcomes, exploratory interaction analyses were conducted using AIS grade, injury level, and cause of injury to evaluate whether treatment responses differed across subgroups. These analyses were not predefined study objectives but were included to enhance the interpretability of the observed patterns.

### Sample size

Accepting a risk of α < 0.05, a power of 90%, and a 20% dropout rate, a total of 57 participants were needed to calculate the effect size of adjusted R^2^= 0.253.

## Results

The study included 57 patients with an average age of 59.60 ± 17.99 years, mostly men (61.4%) (Table 2).

**Table 2:** Clinical and demographic characteristics of the participants

n		57
Gender, n (%)	Female	22 (38.6)
	Male	35 (61.4)
Age		59.60 ± 17.99
American Spinal Injury Association Impairment Scale (AIS), n (%)	A	14 (24.6)
	B	3 (5.3)
	C	12 (21.1)
	D	28 (49.1)
Spinal cord injury level, n (%)	Cervical	13 (22.8)
	Lumbar	17 (29.8)
	Thoracic	27 (47.4)
Cause of injury, n (%)	Infectious-vascular	12 (21.1)
	Oncological	9 (15.8)
	Post-surgery	17 (29.8)
	Traumatic	19 (33.3)
Neurogenic bladder, n (%)	No	14 (24.6)
	Yes	43 (75.4)
Neurogenic intestine, n (%)	No	22 (38.6)
	Yes	35 (61.4)
Delay from injury to program start (days)		18.53 ± 36.84
Number of sessions		39.05 ± 40.91
Functional status, n (%)	Not walking	27 (47.4)
	Walking	30 (52.6)
Months of hospital admission		3.72 ± 3.28

Data expressed with mean ± standard deviation or with relative absolute values (%)

**Fig. 2:**
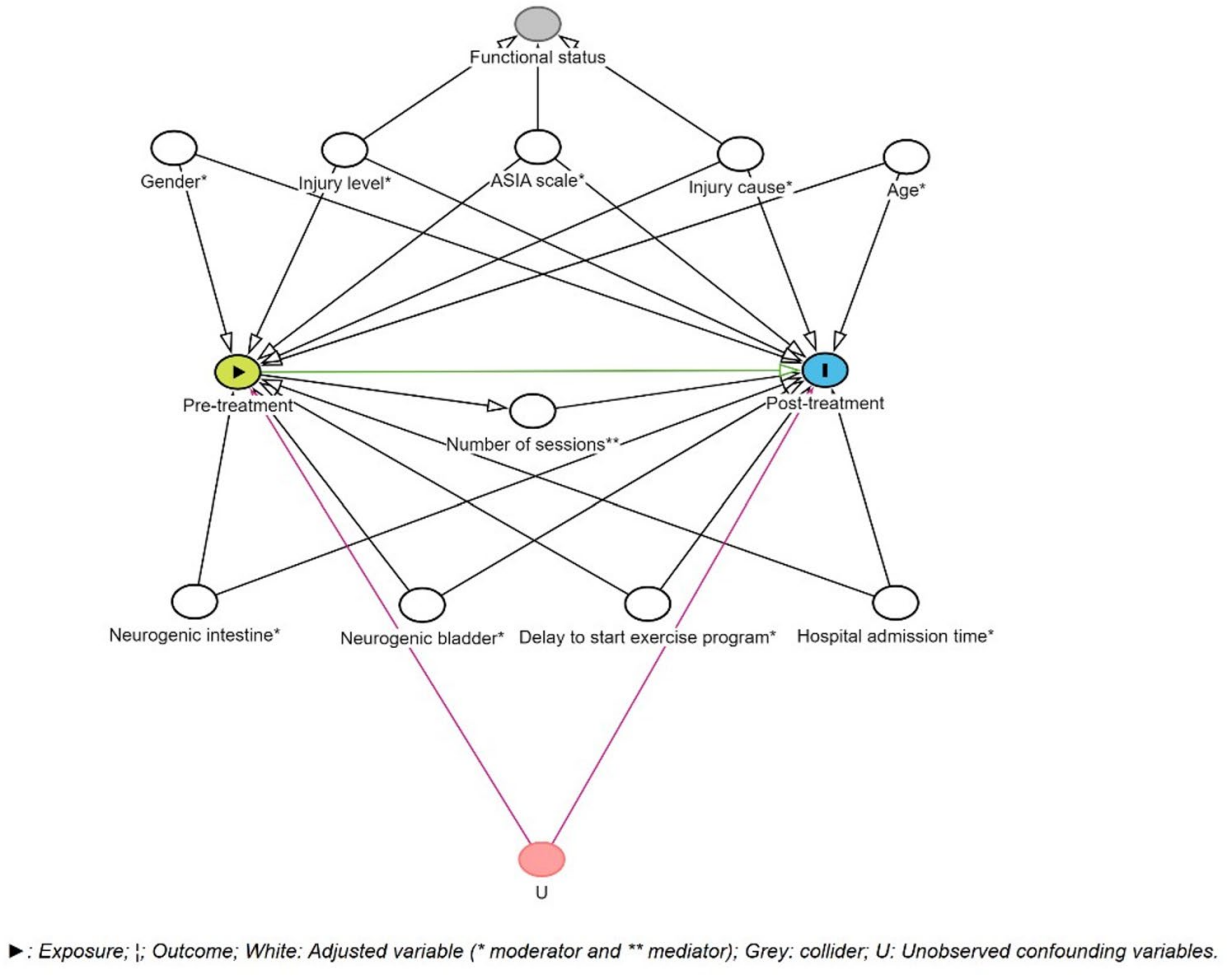
Directed acyclic graph for the effects of pre-treatment values on post-treatment values

The DAG illustrated that the variables to be controlled for the effect of pretreatment values on post-treatment values were sex, age, AIS, spinal cord injury level, cause of injury, neurogenic bladder, neurogenic intestine, delay to start the exercise program, and months of hospital admission as moderators, whereas the number of sessions acted as a mediator (Fig. 2).

We verified that pretreatment values did not significantly affect post-treatment values, either in total or directly, except for the right and left deltoids, right hip abductors, left hip adductors, left knee extensors, and left knee flexors, which remained significant, suggesting the presence of unobserved confounding factors regardless of the number of sessions received (Table 3). For transparency, p-values from permutation-based tests were reported to three decimals; several values were close to 0.10 and were interpreted cautiously given the quasi-experimental design and limited sample size.

**Table 3:** Condensed summary of pre- and post-intervention outcomes and interaction tests

	Post-treatment	Pre-treatment	Average difference post-pretreatment (95%CI)	Total effect (^a^*p* value)	Direct effect (^a^*p* value)	American Spinal Injury Association (AIS) scale: number of sessions (^a^*p* value)	Spinal cord injury level: number of sessions (^a^*p* value)	Cause of injury: number of sessions (^a^*p* value)
Medical Research Council upper-limbs scores								
Overall strength upper-limb right	27.04 ± 6.34	25.58 ± 5.45	4 (3, 5.5)	EDF = 2.065 (dfref = 3), *p* = 0.09	EDF = 2.31 (dfref = 3), *p* = 0.09	X^2^(3) = 13.074, *p* **< 0.001**	X^2^(2) = 1.977, *p* = 0.152	X^2^(3) = 6.763, *p* = **0.001**
Biceps brachii right	4.67 ± 0.99	4.54 ± 0.80	1 (0, 1)	EDF = 2.881 (dfref = 3), *p* = 0.09	EDF = 2.678 (dfref = 3), *p* = 0.09	X^2^(3) = 9.929, *p* **< 0.001**	X^2^(2) = 1.623, *p* = 0.211	X^2^(3) = 3.9, *p* = **0.016**
Deltoids right	4.53 ± 1.07	4.26 ± 1.04	1 (1, 1)	0.783 (SE = 5000), *p***<0.001**	0.782 (SE = 5000), *p* **< 0.001**	X^2^(3) = 16.311, *p* **< 0.001**	X^2^(2) = 0.461, *p* = 0.634	X^2^(3) = 7.111, *p* = **0.001**
Forearm extensors right	4.40 ± 1.31	4.12 ± 1.27	1 (1, 1.5)	EDF = 2.934 (dfref = 3), *p* = 0.09	EDF = 2.973 (dfref = 3), *p* = 0.09	X^2^(3) = 8.95, *p* **< 0.001**	X^2^(2) = 1.637, *p* = 0.208	X^2^(3) = 3.905, *p* = **0.016**
Forearm flexors right	4.44 ± 1.17	4.16 ± 1.18	1 (1, 1.5)	EDF = 2.811 (dfref = 3), *p* = 0.09	EDF = 2.852 (dfref = 3), *p* = 0.09	X^2^(3) = 12.419, *p* **< 0.001**	X^2^(2) = 0.752, *p* = 0.478	X^2^(3) = 5.567, *p* = **0.003**
Latissimus dorsi right	4.51 ± 1.20	4.25 ± 1.06	1 (1, 1.5)	EDF = 1.982 (dfref = 3), *p* = 0.09	EDF = 1.996 (dfref = 3), *p* = 0.09	X^2^(3) = 6.17, *p* = **0.002**	X^2^(2) = 1.048, *p* = 0.36	X^2^(3) = 5.101, *p* = **0.005**
Triceps brachii right	4.49 ± 1.09	4.25 ± 0.99	1 (1, 1)	EDF = 2.052 (dfref = 3), *p* = 0.09	EDF = 2.058 (dfref = 3), *p* = 0.09	X^2^(3) = 9.063, *p* **< 0.001**	X^2^(2) = 2.538, *p* = 0.092	X^2^(3) = 3.476, *p* = **0.025**
Overall strength upper-limb left	27.18 ± 6.21	25.72 ± 5.34	3.5 (2.5, 4.5)	EDF = 2.255 (dfref = 3), *p* = 0.09	EDF = 2.102 (dfref = 3), *p* = 0.09	X^2^(3) = 16.205, *p* **< 0.001**	X^2^(2) = 1.294, *p* = 0.286	X^2^(3) = 9.816, *p* **< 0.001**
Biceps brachii left	4.70 ± 0.96	4.53 ± 0.80	1 (1, 1)	EDF = 2.916 (dfref = 3), *p* = 0.09	EDF = 2.731 (dfref = 3), *p* = 0.09	X^2^(3) = 13.143, *p* **< 0.001**	X^2^(2) = 0.481, *p* = 0.622	X^2^(3) = 5.197, *p* = **0.004**
Deltoids left	4.54 ± 1.02	4.23 ± 1.04	1 (1, 1)	0.693 (SE = 5000), *p* **< 0.001**	0.694 (SE = 5000), *p* **< 0.001**	X^2^(3) = 13.99, *p* **< 0.001**	X^2^(2) = 0.333, *p* = 0.719	X^2^(3) = 5.092, *p* = **0.005**
Forearm extensors left	4.44 ± 1.13	4.25 ± 1.04	1 (0, 1)	EDF = 1.339 (dfref = 3), *p* = 0.09	EDF = 1.597 (dfref = 3), *p* = 0.09	X^2^(3) = 10.206, *p* **< 0.001**	X^2^(2) = 1.139, *p* = 0.331	X^2^(3) = 6.853, *p* = **0.001**
Forearm flexors left	4.46 ± 1.20	4.23 ± 1.13	1 (1, 1)	EDF = 1.771 (dfref = 3), *p* = 0.09	EDF = 1.942 (dfref = 3), *p* = 0.09	X^2^(3) = 11.138, *p* **< 0.001**	X^2^(2) = 1.462, *p* = 0.244	X^2^(3) = 6.798, *p* =** 0.001**
Latissimus dorsi left	4.56 ± 1.17	4.25 ± 1.07	1 (1, 1.5)	EDF = 2.776 (dfref = 3), *p* = 0.09	EDF = 2.795 (dfref = 3), *p* = 0.09	X^2^(3) = 7.218, *p* = **0.001**	X^2^(2) = 0.599, *p* = 0.554	X^2^(3) = 7.14, *p* = **0.001**
Triceps brachii left	4.47 ± 1.14	4.25 ± 0.97	1 (1, 1)	EDF = 1.974 (dfref = 3), *p* = 0.09	EDF = 2.015 (dfref = 3), *p* = 0.09	X^2^(3) = 17.567, *p* **< 0.001**	X^2^(2) = 0.897, *p* = 0.416	X^2^(3) = 10.847, *p* **< 0.001**
Medical Research Council lower limbs scores								
Overall strength lower limbs right	20.54 ± 13.30	16.86 ± 12.54	6.5 (4.5, 8)	0.773 (SE = 0.079), *p* = 0.09	0.884 (SE = 0.079), *p* = 0.09	X^2^(3) = 4.907, *p* = **0.006**	X^2^(2) = 0.366, *p* = 0.696	X^2^(3) = 0.561, *p* = 0.644
Hip abductors right	2.88 ± 1.92	2.30 ± 1.82	1.5 (1, 2)	0.739 (SE = 5000), *p* **< 0.001**	0.818 (SE = 5000), *p* **< 0.001**	X^2^(3) = 3.521, *p* = **0.024**	X^2^(2) = 1.036, *p* = 0.365	X^2^(3) = 0.545, *p* = 0.655
Hip adductors right	2.98 ± 1.93	2.49 ± 1.81	1.5 (1, 2)	0.691 (SE = 0.095), *p* = 0.09	0.802 (SE = 0.099), *p* = 0.09	X^2^(3) = 3.324, *p* = **0.03**	X^2^(2) = 0.475, *p* = 0.625	X^2^(3) = 0.527, *p* = 0.666
Hip flexors right	2.93 ± 1.92	2.26 ± 1.79	1.5 (1.5, 2)	0.752 (SE = 0.087), *p* = 0.09	0.859 (SE = 0.094), *p* = 0.09	X^2^(3) = 3.341, *p* = **0.029**	X^2^(2) = 1.317, *p* = 0.28	X^2^(3) = 0.246, *p* = 0.864
Knee extensors right	3.02 ± 1.95	2.49 ± 1.84	1 (1, 1.5)	0.822 (SE = 0.087), *p* = 0.09	0.879 (SE = 0.085), *p* = 0.09	X^2^(3) = 4.143, *p* = **0.012**	X^2^(2) = 0.996, *p* = 0.379	X^2^(3) = 0.64, *p* = 0.594
Knee flexors right	2.93 ± 1.94	2.33 ± 1.81	1.5 (1, 1.5)	0.801 (SE = 0.089), *p* = 0.09	0.904 (SE = 0.096), *p* = 0.09	X^2^(3) = 2.506, *p* = 0.074	X^2^(2) = 0.374, *p* = 0.69	X^2^(3) = 0.161, *p* = 0.922
Overall strength lower limbs left	19.32 ± 13.15	16.02 ± 12.17	5.5 (4, 7.5)	EDF = 1.99 (dfref = 3), *p* = 0.09	EDF = 1.97 (dfref = 3), *p* = 0.09	X^2^(3) = 2.843, *p* = 0.05	X^2^(2) = 1.222, *p* = 0.306	X^2^(3) = 0.316, *p* = 0.814
Hip abductors left	2.79 ± 1.99	2.32 ± 1.83	1.5 (1, 1.5)	EDF = 0.993 (dfref = 3), *p* = 0.09	EDF = 1.846 (dfref = 3), *p* = 0.09	X^2^(3) = 2.183, *p* = 0.106	X^2^(2) = 1.014, *p* = 0.372	X^2^(3) = 0.25, *p* = 0.861
Hip adductors left	2.95 ± 1.97	2.49 ± 1.84	1 (1, 1.5)	0.725 (SE = 5000), *p* **< 0.001**	0.743 (SE = 5000), *p* **< 0.001**	X^2^(3) = 3.711, *p* = **0.02**	X^2^(2) = 2.497, *p* = 0.096	X^2^(3) = 0.393, *p* = 0.759
Hip flexors left	2.82 ± 1.95	2.25 ± 1.73	1 (1, 1.5)	0.876 (SE = 0.077), *p* = 0.09	0.957 (SE = 0.075), *p* = 0.09	X^2^(3) = 2.779, *p* = 0.054	X^2^(2) = 0.862, *p* = 0.43	X^2^(3) = 0.238, *p* = 0.869
Knee extensors left	2.98 ± 1.93	2.42 ± 1.78	1.5 (1, 1.5)	0.783 (SE = 5000), *p* **< 0.001**	0.833 (SE = 5000), *p* **< 0.001**	X^2^(3) = 6.089, *p* = **0.002**	X^2^(2) = 1.656, *p* = 0.204	X^2^(3) = 1.032, *p* = 0.389
Knee flexors left	2.89 ± 1.91	2.35 ± 1.76	1 (1, 2)	0.713 (SE = 5000), *p* **< 0.001**	0.737 (SE = 5000), *p* **< 0.001**	X^2^(3) = 1.408, *p* = 0.255	X^2^(2) = 0.876, *p* = 0.425	X^2^(3) = 0.16, *p* = 0.922
Functional scores								
6-Minute Walk/Push Test (m)	173.02 ± 153.69	109.25 ± 143.46	87.5 (68, 108)	0.97 (SE = 0.078), *p* = 0.09	1.041 (SE = 0.086), *p* = 0.09	X^2^(3) = 3.153, *p* = **0.036**	X^2^(2) = 0.271, *p* = 0.764	X^2^(3) = 1.352, *p* = 0.273
6-Minute Walk/Push Test assistance level	3.00 ± 3.14	1.89 ± 2.84	4 (3, 5.5)	0.719 (SE = 0.098), *p* = 0.09	0.745 (SE = 0.101), *p* = 0.09	X^2^(3) = 0.591, *p* = 0.625	X^2^(2) = 2.024, *p* = 0.146	X^2^(3) = 2.872, *p* = **0.049**

Post- and pre-treatment values, mean differences with 95% confidence intervals (95% CI), and results of regression models (total and direct effects and interactions by AIS, injury level, and cause of injury). Data are expressed as the mean ± standard deviation. Abbreviations: CI, confidence interval; SE, standard error; EDF, effective degrees of freedom; dfref, reference degrees of freedom. ^*a*^ Significant at *p* < 0.05 (as shown in bold). Note: Full statistical details underpinning these results, including the effective degrees of freedom, multicollinearity assessment, and model fit diagnostics, are provided in the Supplementary Material (Tables S1–S3)

**Fig. 3:**
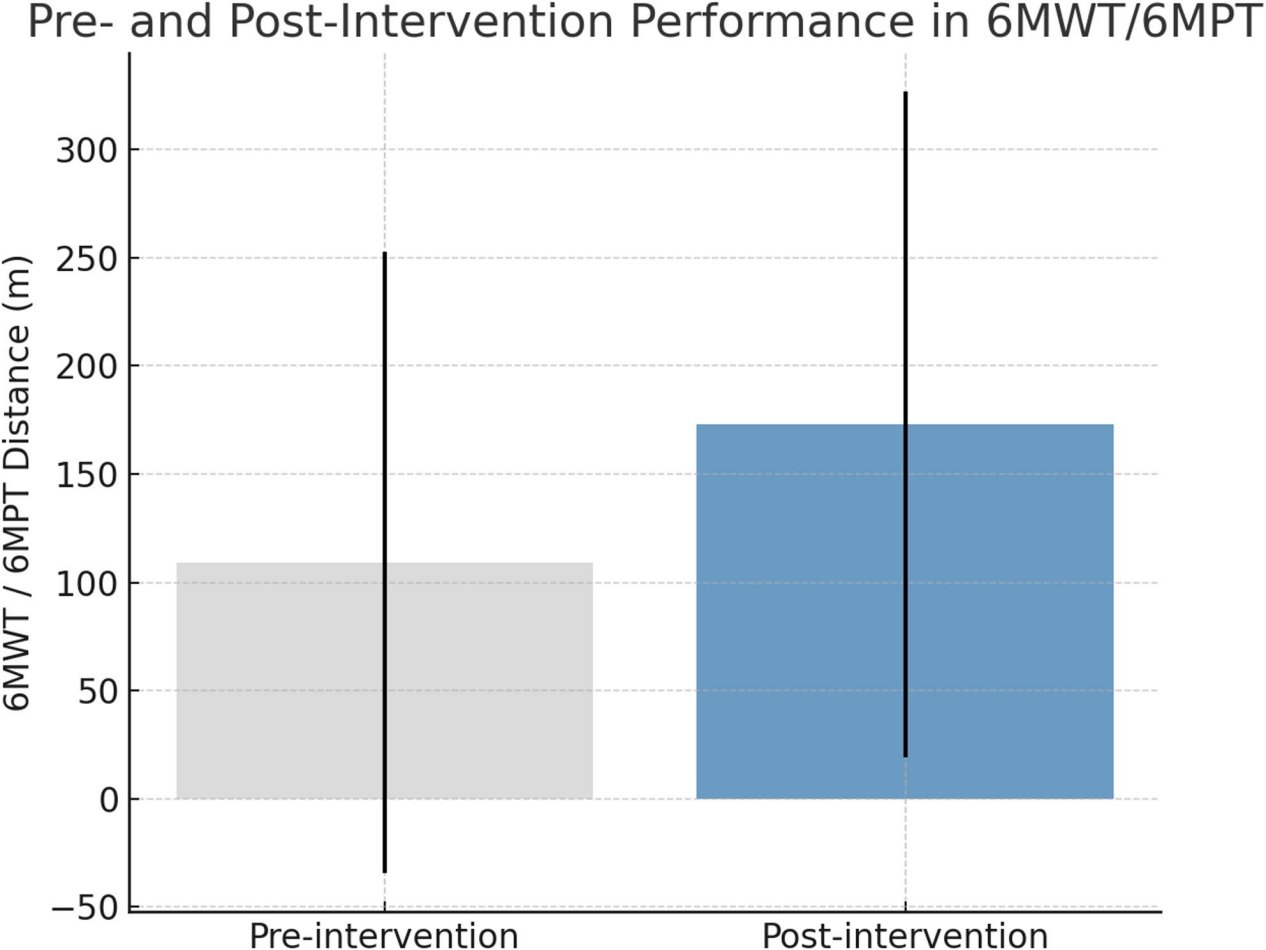
Pre- and post-intervention performance in the 6-Minute Walk Test (6MWT) for ambulatory participants and 6-Minute Push Test (6MPT) for wheelchair users. Data are expressed as mean ± standard deviation

In addition, the mean distance in the 6MWT/6MPT increased significantly from baseline to post-intervention, as illustrated in Fig. 3. This figure shows the pre- and post-program performance distributions, confirming improvements in functional endurance detected in the regression models.

**Fig. 4:**
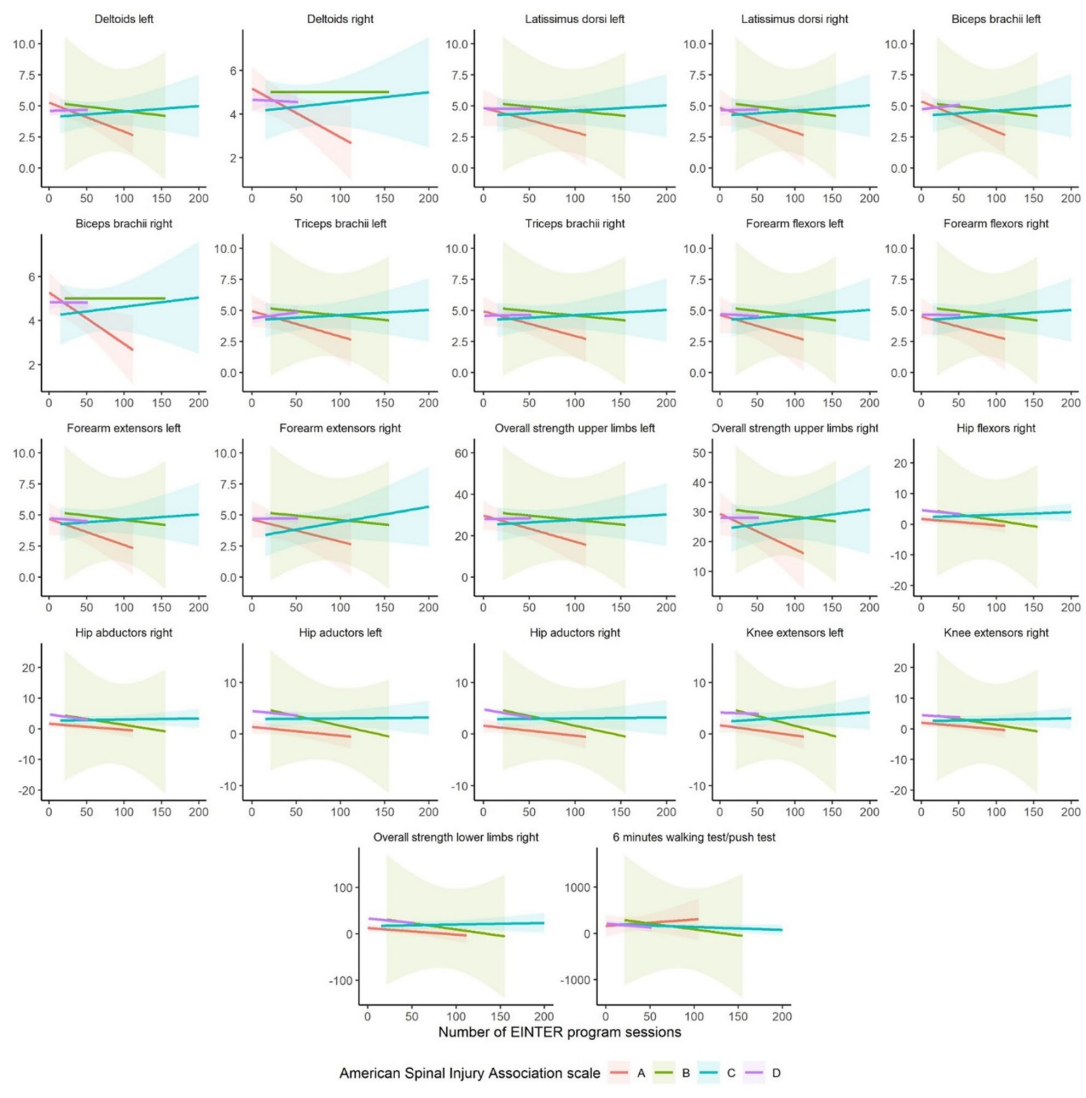
Number of sessions vs. significant post-treatment interaction plots by AIS

**Fig. 5:**
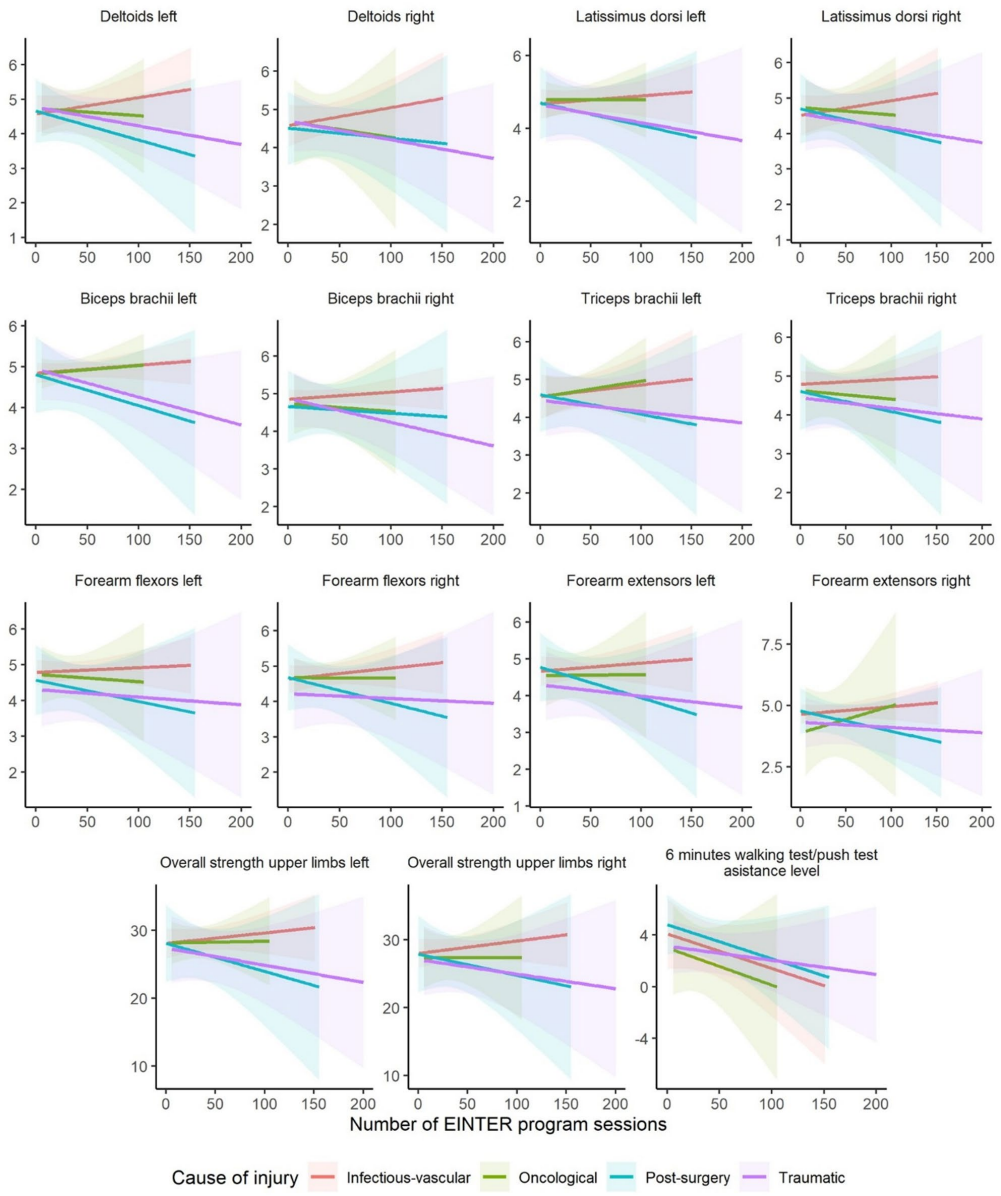
Number of sessions vs. significant post-treatment interaction plots by cause of injury

However, significant post-treatment differences varied according to the number of sessions and the type and cause of injury, systematically for upper-limb muscular strength and less frequently for lower-limb measures. In the functional tests, there was a tendency toward greater improvements with higher session counts among patients with AIS C injuries of infectious–vascular origin; distance in the 6MWT/6MPT increased among patients with AIS A injuries, and those with traumatic injuries required a lower level of assistance (Figs. 4 and 5).

**Fig. 6:**
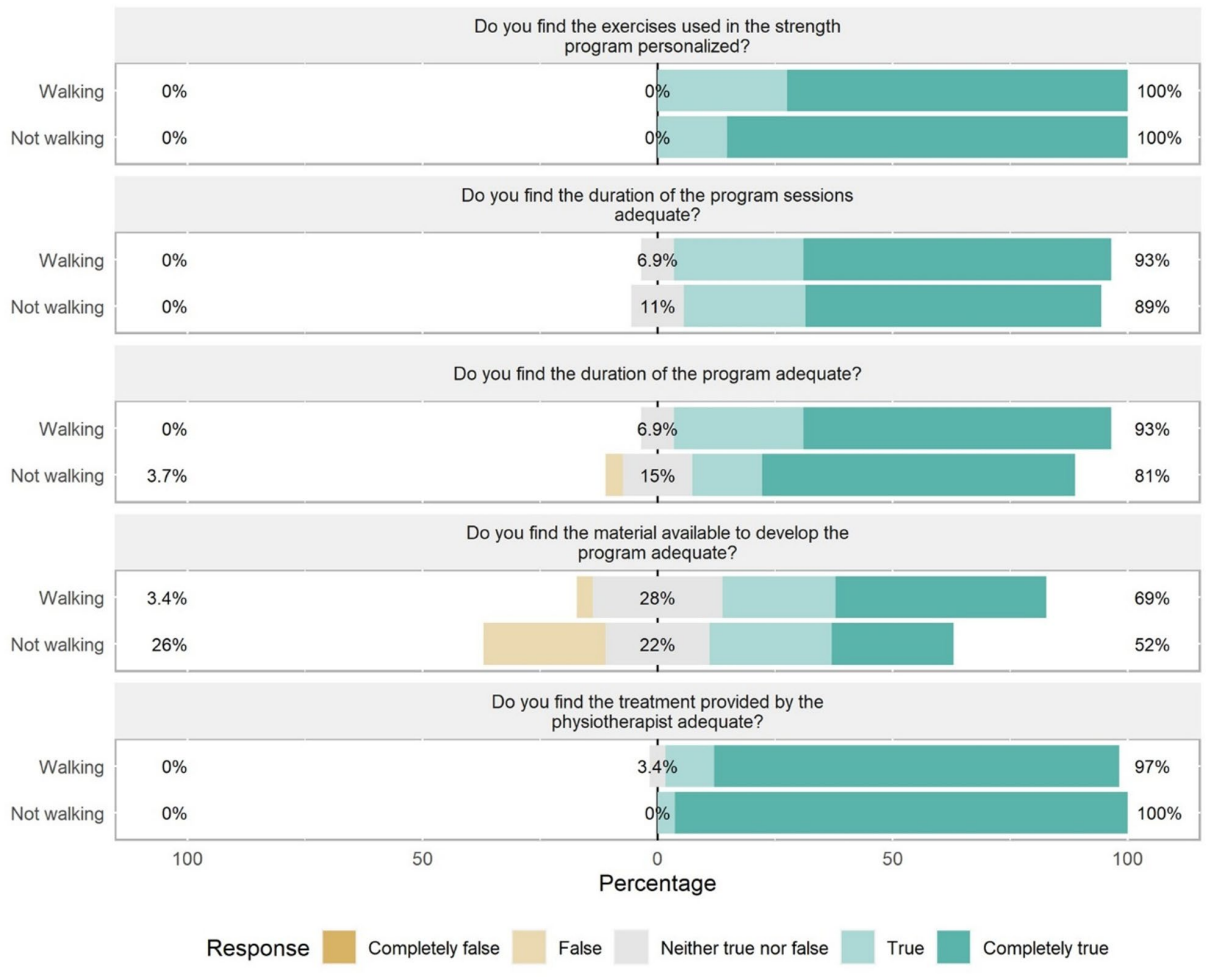
Item response levels

No significant differences were found in satisfaction responses by functional status, with a predominance of positive responses, except for the item “Do you find the material available to develop the program adequate?”, which showed more positive responses in the walking group (4.10 ± 0.94 vs. 3.52 ± 1.16). The non-walking group displayed higher proportions of neutral (25%) and negative (14.29%) responses than positive responses (60.71%) (Table 4; Fig. 6). The distribution of scores shifted toward mid-to-low values (Supplementary Fig. 1), which was reflected in more balanced percentages for “False,” “Neither true nor false,” and “True,” and lower percentages for “Completely true,” which, however, remained the majority for the other items (Supplementary Fig. 2).

**Table 4:** Ad hoc satisfaction questionnaire scores

	Low responses (%)	Neutral responses (%)	High responses (%)	Overall	Not walking group	Walking group	^a^*p* value
n				57	27	30	NA
Do you find the exercises used in the strength program personalized?	0	0	100	4.79 ± 0.41	4.85 ± 0.36	4.72 ± 0.45	0.252
Do you find the duration of the program sessions adequate?	0	8.93	91.07	4.55 ± 0.66	4.52 ± 0.70	4.59 ± 0.63	0.704
Do you find the duration of the program adequate?	1.79	10.71	87.5	4.52 ± 0.76	4.44 ± 0.89	4.59 ± 0.63	0.492
Do you find the material available to develop the program adequate?	14.29	25	60.71	3.82 ± 1.08	3.52 ± 1.16	4.10 ± 0.94	**0.042**
Do you find the treatment provided by the physiotherapist adequate?	0	1.79	98.21	4.89 ± 0.37	4.96 ± 0.19	4.83 ± 0.47	0.168

Data are expressed as the mean ± standard deviation or absolute and relative (%) values. ^a^significant at *p* < 0.05 (shown in bold)

## Discussion

This study investigated the impact of a structured group-based therapeutic exercise program on muscular strength and aerobic capacity in hospitalized patients with spinal cord injury (SCI). The results indicated that participation in the program was associated with improvements, particularly in upper limb strength and functional endurance, within the limits of a single-group design. Our findings further support structured group-based training during inpatient SCI rehabilitation [5, 16]. However, it is important to note that the observed upper limb improvements likely represent two distinct mechanisms. For participants with thoracic or lumbar injuries, the changes are best explained as compensatory strengthening associated with repetitive training and increased use of the unaffected musculature. In contrast, in patients with cervical injuries, gains may partially reflect neurological recovery potential. Recognizing this distinction is essential to avoid over-generalizing the effects of group-based training.

The observed improvements in upper limb strength and endurance may be explained by several mechanisms. In patients with thoracic or lumbar injuries, who typically do not present with direct upper-limb motor deficits, the gains likely reflect compensatory strengthening due to increased reliance on upper-limb function for mobility and daily activities. In contrast, for patients with cervical lesions, improvements could be partially attributed to neuroplastic processes and preserved motor pathways in response to repetitive, progressive loading. Additionally, structured and stratified group training provided consistent stimuli that promoted muscular adaptation through both concentric and eccentric actions, which are well established as effective for increasing strength and endurance in neurological populations. The finding that participants with higher adherence showed more favorable outcomes further supports the role of repeated and progressive training stimuli in driving these adaptations.

Our results were consistent with those of Smith et al. (2023). They observed that both individualized and group-based exercise programs improved activity levels and perceived function in adults with traumatic SCI. This finding highlights the importance of structured supervision and continuity [4, 17]. Similarly, Silva et al. (2025) found that group-based programs led to reductions in pain and disability and improved global functional outcomes in older adults with neuromuscular limitations [18]. Although individual therapy is considered the standard of care for inpatient rehabilitation, group-based models may provide complementary benefits. These include fostering peer support, enhancing motivation, improving adherence, and allowing efficient use of therapeutic resources. Our findings suggest that group training can be a feasible and safe adjunct to routine care, rather than a substitute for individualized therapy.

Beyond physical outcomes, the group-based format may have provided psychosocial benefits that complemented the standard rehabilitation. Exercising alongside peers likely enhances motivation, normalizes the rehabilitation process, and facilitates shared coping strategies. These psychosocial dimensions were reflected in our satisfaction findings, where patients reported high enjoyment and willingness to continue exercising after discharge, although equipment adequacy received lower ratings in non-ambulatory participants. Taken together, these results suggest that group training enriches the rehabilitation experience and may contribute to improved adherence and long-term engagement.

Beyond the effectiveness signals, the intervention demonstrated feasibility within an inpatient neurorehabilitation workflow. Process feasibility was reflected by high attendance and completion across 12 weeks at a 1:4 therapist-to-patient ratio. Safety feasibility was supported by standardized screening, pre/post vital-sign checks, predefined cessation criteria for autonomic instability or musculoskeletal discomfort, and absence of adverse events. Acceptability was indicated by high satisfaction scores, particularly for perceived benefit, enjoyment, and willingness to continue exercising after discharge, whereas lower ratings for equipment adequacy among non-ambulatory participants highlighted a modifiable resource constraint. Collectively, these findings suggest that a supervised, stratified group model can be implemented alongside routine one-to-one therapy with good acceptability and without compromising safety, although resource optimization (e.g., equipment availability for wheelchair users) may further enhance the patient experience.

In contrast to some studies that focused only on ambulatory individuals, our intervention also included nonambulatory patients. We used functionally adapted tools (6MWT and 6MPT) consistent with Gorgey and Dudley (2024) in their high-intensity functional training trial [2, 19]. Functional differentiation based on trunk control further ensures safety, feasibility, and individualization, aligning with Kang and Park’s (2024) recommendations for subgroup-adapted interventions [3, 20].

The greater number of significant differences observed when stratifying outcomes by AIS grade rather than by anatomical injury level likely reflects the fact that AIS captures neurological completeness and functional severity more directly. Patients with the same anatomical level (e.g., thoracic) can present with very different degrees of preserved function, and AIS grading differentiates these profiles. Thus, the ASIA classification provides a more sensitive lens to detect variability in responses to the group-based program.

Beyond the statistical significance, the magnitude of the improvements observed in our study also reached clinical relevance. For the 6MWT, an increase of approximately 50 m has been proposed as the MCID in neurological and SCI populations [21]. Our participants improved by a mean of + 87.5 m, which exceeded this benchmark. Similarly, a 1-point increase in the MRC scale score is generally considered to represent a functionally meaningful change in muscle strength. In our cohort, gains of this magnitude were consistently observed in several upper-limb muscle groups. These results suggest that the improvements were not only statistically detectable but also clinically relevant in terms of functional impact.

Adherence to the structured group-based therapeutic exercise program was high and associated with more favorable outcomes, especially in individuals with incomplete injuries of infectious-vascular origin. This is consistent with Lee and Jeoung’s (2023) finding that adherence to rehabilitation protocols is a key mediator of functional gain in patients with SCI [22]. Additionally, the high satisfaction rates reported in our study reflect previous observations that group-based, socially interactive settings foster motivation and perceived benefits [23].

A notable strength of this study is the integration of robust statistical modeling, including DAG-informed adjustments and generalized additive models. These methods address potential confounders and increase interpretability in quasi-experimental settings, as advocated by Pelletier et al. (2024) [24].

It is important to note that the stratified analyses by cause of injury were exploratory and were not part of the original study objectives. Therefore, these findings should be interpreted with caution and should primarily serve to generate hypotheses for future confirmatory trials.

### Limitations

This single-group quasi-experimental design precludes causal inference, and concurrent routine rehabilitation may have contributed to the observed changes. The sample size, while adequate for internal modelling, limited the power to detect interaction effects. The time since injury was not systematically captured for all participants, constraining the interpretation of subgroup differences by the recovery phase. Heterogeneity in injury level and etiology further introduces residual confounding despite DAG-informed adjustments. For transparency, permutation-based p-values were reported to three decimals; several fell near 0.10, and were interpreted cautiously. Differences in satisfaction with equipment adequacy suggest that material resources may influence perceived benefits and adherence.

Moreover, the study did not assess long-term outcomes, such as community participation, which are essential for evaluating the sustained impact of rehabilitation interventions [25]. Finally, we did not establish minimum LEMS or UEMS thresholds as inclusion criteria; instead, functional safety criteria (trunk control) were used to guide enrollment. This may have contributed to sample heterogeneity, and future trials should consider defining such thresholds to enhance comparability across subgroups.

### Clinical implications and future directions

Taken together, our data indicate that a stratified, group-based program is feasible and acceptable in an inpatient SCI setting, and is associated with improvements in strength and functional endurance. Future multicenter randomized controlled trials should (i) stratify by AIS grade and acute/subacute/chronic phase; (ii) compare group-based plus usual care versus usual care alone and/or individualized therapy; (iii) prespecify co-primary endpoints that include both effectiveness (e.g., MRC, 6MWT/6MPT) and feasibility (safety events, adherence, therapist time, satisfaction, and equipment utilization); and (iv) evaluate durability and cost-effectiveness for implementation.

## Conclusion

In this single-group quasi-experimental study, a supervised, stratified group-based therapeutic exercise program was feasible and acceptable during inpatient SCI rehabilitation, as evidenced by high adherence, high patient satisfaction, and no adverse events, and was associated with preliminary improvements in muscular strength and functional endurance, especially among participants with higher session attendance. Given the absence of a control group, cohort heterogeneity, and concurrent routine rehabilitation, these findings should be considered as exploratory. Well-designed randomized controlled trials, stratified by AIS and time since injury and incorporating prespecified feasibility endpoints (safety, adherence, therapist time, satisfaction, and resource use), are needed to confirm efficacy, assess durability, and determine cost-effectiveness before broad implementation.

## Supplementary Information

Below is the link to the electronic supplementary material.

Supplementary material 1

## Data Availability

Data supporting the findings of this study are available from the corresponding author upon reasonable request. Owing to ethical and legal restrictions, the raw data are not publicly available.

## Abbreviations

SCI: Spinal cord injury
EINTER: Interdisciplinary exercise program
6MWT: 6-minute walk test
6MPT: 6-minute push test
MRC: Medical research council (scale)
DAG: Directed acyclic graph
GAM: Generalized additive model
LM: Linear model
EDF: Effective degrees of freedom
SD: Standard deviation
SE: Standard error
AIS: American Spinal Injury Association Impairment Scale
U.L.M.E.: Spinal cord injury unit (Unidad de Lesionados Medulares)
CI: Confidence interval
R²: Coefficient of determination
NCT: ClinicalTrials.gov Trial Identifier
