# Supplementary material for: Impact and feasibility of a group-based therapeutic exercise program on strength and endurance in hospitalized patients with spinal cord injury: a quasi-experimental study
Source: J Neuroeng Rehabil. 2026 Feb 16;23:113. doi: 10.1186/s12984-025-01845-z (PMC13045115; doi:10.1186/s12984-025-01845-z)

**Supplementary material**

**Supplementary material. Table 1:** Model covariables multicollinearity assesment.

|  |  | ρ |
| --- | --- | --- |
| **Gender** | Age | 0.019 |
|  | American Spinal Injury Association scale | 0.238 |
|  | Spinal cord injury level | 0.225 |
|  | Cause of injury | 0.123 |
|  | Neurogenic bladder | 0.346 |
|  | Neurogenic intestine | 0.184 |
|  | Delay to start exercise program | 0.445 |
|  | Number of sessions | 0.034 |
|  | Months of hospital admission | 0.072 |
| **Age** | Gender | 0.019 |
|  | American Spinal Injury Association scale | 0.189 |
|  | Spinal cord injury level | 0.185 |
|  | Cause of injury | 0.261 |
|  | Neurogenic bladder | 0.062 |
|  | Neurogenic intestine | 0.008 |
|  | Delay to start exercise program | 0.105 |
|  | Number of sessions | 0.098 |
|  | Months of hospital admission | 0.067 |
| **American Spinal Injury Association scale** | Gender | 0.238 |
|  | Age | 0.189 |
|  | Spinal cord injury level | 0.331 |
|  | Cause of injury | 0.343 |
|  | Neurogenic bladder | 0.912 |
|  | Neurogenic intestine | 0.816 |
|  | Delay to start exercise program | 0.274 |
|  | Number of sessions | 0.263 |
|  | Months of hospital admission | 0.529 |
| **Spinal cord injury level** | Gender | 0.225 |
|  | Age | 0.185 |
|  | American Spinal Injury Association scale | 0.331 |
|  | Cause of injury | 0.052 |
|  | Neurogenic bladder | 0.509 |
|  | Neurogenic intestine | 0.363 |
|  | Delay to start exercise program | 0.539 |
|  | Number of sessions | 0.296 |
|  | Months of hospital admission | 0.173 |
| **Cause of injury** | Gender | 0.123 |
|  | Age | 0.261 |
|  | American Spinal Injury Association scale | 0.343 |
|  | Spinal cord injury level | 0.052 |
|  | Neurogenic bladder | 0.097 |
|  | Neurogenic intestine | 0.089 |
|  | Delay to start exercise program | 0.005 |
|  | Number of sessions | 0.111 |
|  | Months of hospital admission | 0.16 |
| **Neurogenic bladder** | Gender | 0.346 |
|  | Age | 0.062 |
|  | American Spinal Injury Association scale | 0.912 |
|  | Spinal cord injury level | 0.509 |
|  | Cause of injury | 0.097 |
|  | Neurogenic intestine | 0.924 |
|  | Delay to start exercise program | 0.347 |
|  | Number of sessions | 0.377 |
|  | Months of hospital admission | 0.558 |
| **Neurogenic intestine** | Gender | 0.184 |
|  | Age | 0.008 |
|  | American Spinal Injury Association scale | 0.816 |
|  | Spinal cord injury level | 0.363 |
|  | Cause of injury | 0.089 |
|  | Neurogenic bladder | 0.924 |
|  | Delay to start exercise program | 0.427 |
|  | Number of sessions | 0.26 |
|  | Months of hospital admission | 0.435 |
| **Delay to start exercise program** | Gender | 0.445 |
|  | Age | 0.105 |
|  | American Spinal Injury Association scale | 0.274 |
|  | Spinal cord injury level | 0.539 |
|  | Cause of injury | 0.005 |
|  | Neurogenic bladder | 0.347 |
|  | Neurogenic intestine | 0.427 |
|  | Number of sessions | 0.049 |
|  | Months of hospital admission | 0.354 |
| **Number of sessions** | Gender | 0.034 |
|  | Age | 0.098 |
|  | American Spinal Injury Association scale | 0.263 |
|  | Spinal cord injury level | 0.296 |
|  | Cause of injury | 0.111 |
|  | Neurogenic bladder | 0.377 |
|  | Neurogenic intestine | 0.26 |
|  | Delay to start exercise program | 0.049 |
|  | Months of hospital admission | 0.572 |
| **Months of hospital admission** | Gender | 0.072 |
|  | Age | 0.067 |
|  | American Spinal Injury Association scale | 0.529 |
|  | Spinal cord injury level | 0.173 |
|  | Cause of injury | 0.16 |
|  | Neurogenic bladder | 0.558 |
|  | Neurogenic intestine | 0.435 |
|  | Delay to start exercise program | 0.354 |
|  | Number of sessions | 0.572 |

Correlations higher than 0.7 are shown in red.

**Supplementary material. Table 2:** Model covariables effective degrees of freedom.

|  | **Pre-treatment values** | **Number of sessions** | **Age** | **Delay to start exercise program (days)** | **Months of hospital admission** |
| --- | --- | --- | --- | --- | --- |
| Deltoids left | 1.055 | 0.644 | 0.524 | 0 | 1.827 |
| Deltoids right | 1.329 | 1.019 | 0.523 | 0 | 1.959 |
| Latissimus dorsi left | 2.87 | 1.338 | 0.688 | 0 | 2.22 |
| Latissimus dorsi right | 2.053 | 1.466 | 0.463 | 1.395 | 2.955 |
| Biceps brachii left | 2.781 | 2.861 | 0 | 0.683 | 2.9 |
| Biceps brachii right | 2.764 | 2.752 | 0.346 | 1.368 | 2.906 |
| Triceps brachii left | 2.003 | 2.088 | 0.562 | 0.852 | 2.982 |
| Triceps brachii right | 2.071 | 1.53 | 0.324 | 1.335 | 2.92 |
| Forearm flexors left | 2.081 | 2.867 | 0 | 0.942 | 2.842 |
| Forearm flexors right | 2.75 | 2.871 | 0 | 0.833 | 2.935 |
| Forearm extensors left | 2.695 | 1.635 | 0.499 | 0.899 | 2.92 |
| Forearm extensors right | 2.887 | 2.842 | 0 | 0.804 | 2.89 |
| Overall strength upper limbs left | 2.141 | 2.824 | 0.324 | 0.862 | 2.929 |
| Overall strength upper limbs right | 2.122 | 2.853 | 0 | 0.878 | 2.778 |
| Hip flexors left | 1.925 | 2.671 | 0 | 0 | 0 |
| Hip flexors right | 1.518 | 2.807 | 0 | 0 | 0 |
| Hip abductors left | 2.044 | 2.783 | 0 | 0 | 0 |
| Hip abductors right | 1.007 | 0 | 0.539 | 0 | 0 |
| Hip aductors left | 0.976 | 0.582 | 0.083 | 0 | 0 |
| Hip aductors right | 1 | 2.858 | 1.537 | 0 | 0 |
| Knee flexors left | 0.951 | 0 | 0.048 | 0.895 | 0 |
| Knee flexors right | 0.997 | 2.849 | 0 | 0 | 0 |
| Knee extensors left | 0.988 | 0 | 0 | 0 | 0 |
| Knee extensors right | 0.996 | 2.928 | 0 | 0.036 | 0 |
| Ankle flexors left | 0.997 | 0 | 0.854 | 1.342 | 1.668 |
| Ankle flexors right | 2.091 | 2.821 | 1.746 | 0 | 0 |
| Ankle extensors left | 0.977 | 0 | 1.724 | 0.869 | 0 |
| Ankle extensors right | 1 | 2.955 | 1.64 | 0 | 0 |
| Overall strength lower limbs left | 2.012 | 0 | 0.13 | 0.947 | 0 |
| Overall strength lower limbs right | 1 | 2.902 | 1.377 | 0 | 0 |
| 6 minutes walking test/push test (mtrs) | 2.582 | 0.687 | 1.375 | 1.545 | 2.787 |
| 6 minutes walking test/push test asistance level | 0.982 | 0 | 0.605 | 0 | 0 |

Values higher than 2 are shown in red.

**Supplementary material. Table 3:** Models fit assesment.

|  | | | **GAM models pairwise concurvity*** | | |  | **GAM models residuals assesment** | | | **LM models residuals assesment** | | |
| --- | --- | --- | --- | --- | --- | --- | --- | --- | --- | --- | --- | --- |
|  |  | **GAM models global concurvity*** | **Pre-treatment** | **Number of sessions** | **Age** | **K index (^a^p value)** | **Kolmogorov-Smirnov test (^a^p value)** | **Breusch-Pagan test (^a^p value)** | **Durbin-Watson test (^a^p value)** | **Kolmogorov-Smirnov test (^a^p value)** | **Breusch-Pagan test (^a^p value)** | **Durbin-Watson test (^a^p value)** |
| **Deltoids left** | Pre-treatment |  |  |  |  |  |  |  |  | <0.001 | <0.001 | 0.266 |
|  | Number of sessions |  |  |  |  |  |  |  |  | <0.001 | <0.001 | 0.266 |
|  | Months of hospital admission |  |  |  |  |  |  |  |  | <0.001 | <0.001 | 0.266 |
|  | Delay to start exercise program |  |  |  |  |  |  |  |  | <0.001 | <0.001 | 0.266 |
|  | Age |  |  |  |  |  |  |  |  | <0.001 | <0.001 | 0.266 |
| **Deltoids right** | Pre-treatment |  |  |  |  |  |  |  |  | 0.002 | <0.001 | 0.136 |
|  | Number of sessions |  |  |  |  |  |  |  |  | 0.002 | <0.001 | 0.136 |
|  | Months of hospital admission |  |  |  |  |  |  |  |  | 0.002 | <0.001 | 0.136 |
|  | Delay to start exercise program |  |  |  |  |  |  |  |  | 0.002 | <0.001 | 0.136 |
|  | Age |  |  |  |  |  |  |  |  | 0.002 | <0.001 | 0.136 |
| **Latissimus dorsi left** | Pre-treatment | 0.195 | 1 | 0.035 | 0.037 | 1.113, p=0.782 | 0.004 | <0.001 | 0.714 |  |  |  |
|  | Number of sessions | 0.628 | 0.023 | 1 | 0.04 | 1.128, p=0.8 | 0.004 | <0.001 | 0.714 |  |  |  |
|  | Months of hospital admission | 0.541 | 0.003 | 0.398 | 0.092 | 1.349, p=>0.999 | 0.004 | <0.001 | 0.714 |  |  |  |
|  | Delay to start exercise program | 0.494 | 0.021 | 0.034 | 0.015 | 0.932, p=0.24 | 0.004 | <0.001 | 0.714 |  |  |  |
|  | Age | 0.319 | 0.031 | 0.035 | 1 | 1.169, p=0.882 | 0.004 | <0.001 | 0.714 |  |  |  |
| **Latissimus dorsi right** | Pre-treatment | 0.18 | 1 | 0.004 | 0.067 | 0.96, p=0.298 | 0.019 | <0.001 | 0.948 |  |  |  |
|  | Number of sessions | 0.621 | 0.017 | 1 | 0.039 | 1.134, p=0.84 | 0.019 | <0.001 | 0.948 |  |  |  |
|  | Months of hospital admission | 0.542 | 0.013 | 0.403 | 0.082 | 1.34, p=0.998 | 0.019 | <0.001 | 0.948 |  |  |  |
|  | Delay to start exercise program | 0.572 | 0.005 | 0.035 | 0.015 | 0.923, p=0.252 | 0.019 | <0.001 | 0.948 |  |  |  |
|  | Age | 0.316 | 0.036 | 0.035 | 1 | 1.17, p=0.905 | 0.019 | <0.001 | 0.948 |  |  |  |
| **Biceps brachii left** | Pre-treatment | 0.297 | 1 | 0.021 | 0.047 | 1.241, p=0.955 | <0.001 | <0.001 | 0.072 |  |  |  |
|  | Number of sessions | 0.601 | 0.034 | 1 | 0.039 | 1.028, p=0.562 | <0.001 | <0.001 | 0.072 |  |  |  |
|  | Months of hospital admission | 0.501 | 0.056 | 0.342 | 0.082 | 1.324, p=0.988 | <0.001 | <0.001 | 0.072 |  |  |  |
|  | Delay to start exercise program | 0.656 | 0.021 | 0.051 | 0.015 | 1.121, p=0.79 | <0.001 | <0.001 | 0.072 |  |  |  |
|  | Age | 0.285 | 0.054 | 0.038 | 1 | 1.359, p=0.99 | <0.001 | <0.001 | 0.072 |  |  |  |
| **Biceps brachii right** | Pre-treatment | 0.298 | 1 | 0.01 | 0.043 | 1.291, p=0.98 | 0.092 | <0.001 | 0.468 |  |  |  |
|  | Number of sessions | 0.548 | 0.04 | 1 | 0.039 | 0.983, p=0.372 | 0.092 | <0.001 | 0.468 |  |  |  |
|  | Months of hospital admission | 0.506 | 0.047 | 0.313 | 0.082 | 1.344, p=0.998 | 0.092 | <0.001 | 0.468 |  |  |  |
|  | Delay to start exercise program | 0.631 | 0.018 | 0.045 | 0.015 | 1.019, p=0.502 | 0.092 | <0.001 | 0.468 |  |  |  |
|  | Age | 0.288 | 0.059 | 0.034 | 1 | 1.354, p=0.995 | 0.092 | <0.001 | 0.468 |  |  |  |
| **Triceps brachii left** | Pre-treatment | 0.212 | 1 | 0.039 | 0.016 | 1.192, p=0.875 | 0.01 | <0.001 | 0.072 |  |  |  |
|  | Number of sessions | 0.663 | 0.025 | 1 | 0.039 | 1.002, p=0.478 | 0.01 | <0.001 | 0.072 |  |  |  |
|  | Months of hospital admission | 0.661 | 0.014 | 0.407 | 0.082 | 1.515, p=>0.999 | 0.01 | <0.001 | 0.072 |  |  |  |
|  | Delay to start exercise program | 0.634 | 0.015 | 0.054 | 0.015 | 0.975, p=0.365 | 0.01 | <0.001 | 0.072 |  |  |  |
|  | Age | 0.298 | 0.048 | 0.045 | 1 | 1.248, p=0.965 | 0.01 | <0.001 | 0.072 |  |  |  |
| **Triceps brachii right** | Pre-treatment | 0.236 | 1 | 0.019 | 0.039 | 1.086, p=0.75 | 0.001 | <0.001 | 0.95 |  |  |  |
|  | Number of sessions | 0.658 | 0.046 | 1 | 0.045 | 0.969, p=0.328 | 0.001 | <0.001 | 0.95 |  |  |  |
|  | Months of hospital admission | 0.601 | 0.011 | 0.432 | 0.172 | 1.332, p=0.998 | 0.001 | <0.001 | 0.95 |  |  |  |
|  | Delay to start exercise program | 0.437 | 0.006 | 0.037 | 0.018 | 0.949, p=0.248 | 0.001 | <0.001 | 0.95 |  |  |  |
|  | Age | 0.397 | 0.075 | 0.036 | 1 | 1.144, p=0.84 | 0.001 | <0.001 | 0.95 |  |  |  |
| **Forearm flexors left** | Pre-treatment | 0.207 | 1 | 0.014 | 0.009 | 1.125, p=0.815 | 0.157 | <0.001 | 0.926 |  |  |  |
|  | Number of sessions | 0.638 | 0.037 | 1 | 0.039 | 1.076, p=0.695 | 0.157 | <0.001 | 0.926 |  |  |  |
|  | Months of hospital admission | 0.611 | 0.008 | 0.38 | 0.082 | 1.358, p=0.998 | 0.157 | <0.001 | 0.926 |  |  |  |
|  | Delay to start exercise program | 0.624 | 0.004 | 0.052 | 0.015 | 0.936, p=0.255 | 0.157 | <0.001 | 0.926 |  |  |  |
|  | Age | 0.272 | 0.045 | 0.04 | 1 | 1.306, p=0.985 | 0.157 | <0.001 | 0.926 |  |  |  |
| **Forearm flexors right** | Pre-treatment | 0.193 | 1 | 0.014 | 0.022 | 0.995, p=0.375 | 0.042 | <0.001 | 0.78 |  |  |  |
|  | Number of sessions | 0.659 | 0.028 | 1 | 0.047 | 1.019, p=0.492 | 0.042 | <0.001 | 0.78 |  |  |  |
|  | Months of hospital admission | 0.513 | 0.003 | 0.432 | 0.161 | 1.366, p=>0.999 | 0.042 | <0.001 | 0.78 |  |  |  |
|  | Delay to start exercise program | 0.619 | 0.004 | 0.037 | 0.017 | 0.845, p=0.082 | 0.042 | <0.001 | 0.78 |  |  |  |
|  | Age | 0.386 | 0.053 | 0.036 | 1 | 1.183, p=0.925 | 0.042 | <0.001 | 0.78 |  |  |  |
| **Forearm extensors left** | Pre-treatment | 0.172 | 1 | 0.003 | 0.009 | 1.199, p=0.935 | 0.145 | <0.001 | 0.86 |  |  |  |
|  | Number of sessions | 0.674 | 0.037 | 1 | 0.039 | 1.002, p=0.492 | 0.145 | <0.001 | 0.86 |  |  |  |
|  | Months of hospital admission | 0.575 | 0.014 | 0.416 | 0.082 | 1.241, p=0.958 | 0.145 | <0.001 | 0.86 |  |  |  |
|  | Delay to start exercise program | 0.508 | 0.003 | 0.036 | 0.015 | 0.815, p=0.045 | 0.145 | <0.001 | 0.86 |  |  |  |
|  | Age | 0.26 | 0.035 | 0.035 | 1 | 1.249, p=0.975 | 0.145 | <0.001 | 0.86 |  |  |  |
| **Forearm extensors right** | Pre-treatment | 0.298 | 1 | 0.013 | 0.033 | 1.115, p=0.782 | 0.064 | <0.001 | 0.724 |  |  |  |
|  | Number of sessions | 0.686 | 0.014 | 1 | 0.046 | 0.977, p=0.378 | 0.064 | <0.001 | 0.724 |  |  |  |
|  | Months of hospital admission | 0.522 | 0.009 | 0.428 | 0.16 | 1.281, p=0.975 | 0.064 | <0.001 | 0.724 |  |  |  |
|  | Delay to start exercise program | 0.638 | 0 | 0.037 | 0.017 | 0.937, p=0.24 | 0.064 | <0.001 | 0.724 |  |  |  |
|  | Age | 0.381 | 0.056 | 0.036 | 1 | 1.078, p=0.665 | 0.064 | <0.001 | 0.724 |  |  |  |
| **Overall strength upper limbs left** | Pre-treatment | 0.203 | 1 | 0.007 | 0.02 | 1.122, p=0.78 | 0.114 | <0.001 | 0.114 |  |  |  |
|  | Number of sessions | 0.647 | 0.041 | 1 | 0.039 | 1.041, p=0.542 | 0.114 | <0.001 | 0.114 |  |  |  |
|  | Months of hospital admission | 0.61 | 0.007 | 0.38 | 0.082 | 1.463, p=>0.999 | 0.114 | <0.001 | 0.114 |  |  |  |
|  | Delay to start exercise program | 0.638 | 0.021 | 0.051 | 0.015 | 0.954, p=0.322 | 0.114 | <0.001 | 0.114 |  |  |  |
|  | Age | 0.267 | 0.046 | 0.04 | 1 | 1.357, p=>0.999 | 0.114 | <0.001 | 0.114 |  |  |  |
| **Overall strength upper limbs right** | Pre-treatment | 0.215 | 1 | 0.009 | 0.043 | 1.075, p=0.688 | 0.001 | <0.001 | 0.788 |  |  |  |
|  | Number of sessions | 0.608 | 0.043 | 1 | 0.039 | 1.071, p=0.685 | 0.001 | <0.001 | 0.788 |  |  |  |
|  | Months of hospital admission | 0.602 | 0.011 | 0.398 | 0.087 | 1.35, p=0.995 | 0.001 | <0.001 | 0.788 |  |  |  |
|  | Delay to start exercise program | 0.522 | 0.007 | 0.034 | 0.015 | 0.948, p=0.26 | 0.001 | <0.001 | 0.788 |  |  |  |
|  | Age | 0.272 | 0.064 | 0.035 | 1 | 1.185, p=0.922 | 0.001 | <0.001 | 0.788 |  |  |  |
| **Hip flexors left** | Pre-treatment | 0.621 | 1 | 0.081 | 0.077 | 0.912, p=0.2 | 0.776 | 0.419 | 0.588 |  |  |  |
|  | Number of sessions | 0.53 | 0.214 | 1 | 0.041 | 1.04, p=0.568 | 0.776 | 0.419 | 0.588 |  |  |  |
|  | Months of hospital admission | 0.674 | 0.149 | 0.196 | 0.099 | 1.245, p=0.96 | 0.776 | 0.419 | 0.588 |  |  |  |
|  | Delay to start exercise program | 0.332 | 0.149 | 0.005 | 0.015 | 1.034, p=0.532 | 0.776 | 0.419 | 0.588 |  |  |  |
|  | Age | 0.307 | 0.031 | 0.032 | 1 | 0.762, p=0.015 | 0.776 | 0.419 | 0.588 |  |  |  |
| **Hip flexors right** | Pre-treatment | 0.703 | 1 | 0.148 | 0.052 | 0.897, p=0.192 | 0.091 | 0.979 | 0.112 |  |  |  |
|  | Number of sessions | 0.581 | 0.31 | 1 | 0.039 | 1.118, p=0.723 | 0.091 | 0.979 | 0.112 |  |  |  |
|  | Months of hospital admission | 0.68 | 0.221 | 0.24 | 0.082 | 1.062, p=0.657 | 0.091 | 0.979 | 0.112 |  |  |  |
|  | Delay to start exercise program | 0.606 | 0.183 | 0.009 | 0.015 | 1.159, p=0.868 | 0.091 | 0.979 | 0.112 |  |  |  |
|  | Age | 0.254 | 0.013 | 0.026 | 1 | 0.869, p=0.12 | 0.091 | 0.979 | 0.112 |  |  |  |
| **Hip abductors left** | Pre-treatment | 0.602 | 1 | 0.04 | 0.065 | 1.063, p=0.618 | 0.137 | 0.254 | 0.926 |  |  |  |
|  | Number of sessions | 0.399 | 0.186 | 1 | 0.039 | 1.122, p=0.797 | 0.137 | 0.254 | 0.926 |  |  |  |
|  | Months of hospital admission | 0.69 | 0.133 | 0.035 | 0.091 | 1.08, p=0.66 | 0.137 | 0.254 | 0.926 |  |  |  |
|  | Delay to start exercise program | 0.359 | 0.161 | 0.012 | 0.016 | 1.061, p=0.578 | 0.137 | 0.254 | 0.926 |  |  |  |
|  | Age | 0.305 | 0.025 | 0.011 | 1 | 0.826, p=0.068 | 0.137 | 0.254 | 0.926 |  |  |  |
| **Hip abductors right** | Pre-treatment |  |  |  |  |  |  |  |  | 0.706 | 0.779 | 0.544 |
|  | Number of sessions |  |  |  |  |  |  |  |  | 0.706 | 0.779 | 0.544 |
|  | Months of hospital admission |  |  |  |  |  |  |  |  | 0.706 | 0.779 | 0.544 |
|  | Delay to start exercise program |  |  |  |  |  |  |  |  | 0.706 | 0.779 | 0.544 |
|  | Age |  |  |  |  |  |  |  |  | 0.706 | 0.779 | 0.544 |
| **Hip aductors left** | Pre-treatment |  |  |  |  |  |  |  |  | 0.22 | 0.004 | 0.56 |
|  | Number of sessions |  |  |  |  |  |  |  |  | 0.22 | 0.004 | 0.56 |
|  | Months of hospital admission |  |  |  |  |  |  |  |  | 0.22 | 0.004 | 0.56 |
|  | Delay to start exercise program |  |  |  |  |  |  |  |  | 0.22 | 0.004 | 0.56 |
|  | Age |  |  |  |  |  |  |  |  | 0.22 | 0.004 | 0.56 |
| **Hip aductors right** | Pre-treatment | 0.657 | 1 | 0.048 | 0.086 | 1.005, p=0.46 | 0.12 | 0.035 | 0.68 |  |  |  |
|  | Number of sessions | 0.378 | 0.24 | 1 | 0.06 | 1.037, p=0.545 | 0.12 | 0.035 | 0.68 |  |  |  |
|  | Months of hospital admission | 0.658 | 0.18 | 0.019 | 0.124 | 0.907, p=0.21 | 0.12 | 0.035 | 0.68 |  |  |  |
|  | Delay to start exercise program | 0.492 | 0.115 | 0.017 | 0.013 | 0.95, p=0.3 | 0.12 | 0.035 | 0.68 |  |  |  |
|  | Age | 0.331 | 0.024 | 0.008 | 1 | 0.978, p=0.408 | 0.12 | 0.035 | 0.68 |  |  |  |
| **Knee flexors left** | Pre-treatment |  |  |  |  |  |  |  |  | 0.084 | 0.018 | 0.592 |
|  | Number of sessions |  |  |  |  |  |  |  |  | 0.084 | 0.018 | 0.592 |
|  | Months of hospital admission |  |  |  |  |  |  |  |  | 0.084 | 0.018 | 0.592 |
|  | Delay to start exercise program |  |  |  |  |  |  |  |  | 0.084 | 0.018 | 0.592 |
|  | Age |  |  |  |  |  |  |  |  | 0.084 | 0.018 | 0.592 |
| **Knee flexors right** | Pre-treatment | 0.695 | 1 | 0.145 | 0.012 | 1.14, p=0.815 | 0.147 | 0.154 | 0.298 |  |  |  |
|  | Number of sessions | 0.512 | 0.281 | 1 | 0.043 | 1.18, p=0.89 | 0.147 | 0.154 | 0.298 |  |  |  |
|  | Months of hospital admission | 0.623 | 0.282 | 0.174 | 0.058 | 0.819, p=0.052 | 0.147 | 0.154 | 0.298 |  |  |  |
|  | Delay to start exercise program | 0.391 | 0.178 | 0.01 | 0.018 | 1.064, p=0.637 | 0.147 | 0.154 | 0.298 |  |  |  |
|  | Age | 0.213 | 0.021 | 0.014 | 1 | 0.977, p=0.39 | 0.147 | 0.154 | 0.298 |  |  |  |
| **Knee extensors left** | Pre-treatment |  |  |  |  |  |  |  |  | 0.065 | 0.001 | 0.076 |
|  | Number of sessions |  |  |  |  |  |  |  |  | 0.065 | 0.001 | 0.076 |
|  | Months of hospital admission |  |  |  |  |  |  |  |  | 0.065 | 0.001 | 0.076 |
|  | Delay to start exercise program |  |  |  |  |  |  |  |  | 0.065 | 0.001 | 0.076 |
|  | Age |  |  |  |  |  |  |  |  | 0.065 | 0.001 | 0.076 |
| **Knee extensors right** | Pre-treatment | 0.618 | 1 | 0.053 | 0.009 | 1.121, p=0.772 | 0.878 | 0.005 | 0.264 |  |  |  |
|  | Number of sessions | 0.345 | 0.222 | 1 | 0.041 | 1.098, p=0.725 | 0.878 | 0.005 | 0.264 |  |  |  |
|  | Months of hospital admission | 0.622 | 0.19 | 0.022 | 0.056 | 0.941, p=0.282 | 0.878 | 0.005 | 0.264 |  |  |  |
|  | Delay to start exercise program | 0.476 | 0.1 | 0.018 | 0.018 | 0.694, p=0.015 | 0.878 | 0.005 | 0.264 |  |  |  |
|  | Age | 0.218 | 0.025 | 0.002 | 1 | 0.906, p=0.168 | 0.878 | 0.005 | 0.264 |  |  |  |
| **Ankle flexors left** | Pre-treatment |  |  |  |  |  |  |  |  | 0.28 | 0.979 | 0.754 |
|  | Number of sessions |  |  |  |  |  |  |  |  | 0.28 | 0.979 | 0.754 |
|  | Months of hospital admission |  |  |  |  |  |  |  |  | 0.28 | 0.979 | 0.754 |
|  | Delay to start exercise program |  |  |  |  |  |  |  |  | 0.28 | 0.979 | 0.754 |
|  | Age |  |  |  |  |  |  |  |  | 0.28 | 0.979 | 0.754 |
| **Ankle flexors right** | Pre-treatment | 0.646 | 1 | 0.108 | 0.024 | 1.151, p=0.845 | 0.297 | 0.245 | 0.09 |  |  |  |
|  | Number of sessions | 0.494 | 0.264 | 1 | 0.034 | 0.982, p=0.415 | 0.297 | 0.245 | 0.09 |  |  |  |
|  | Months of hospital admission | 0.662 | 0.231 | 0.194 | 0.047 | 0.863, p=0.128 | 0.297 | 0.245 | 0.09 |  |  |  |
|  | Delay to start exercise program | 0.383 | 0.114 | 0.008 | 0.014 | 1.181, p=0.882 | 0.297 | 0.245 | 0.09 |  |  |  |
|  | Age | 0.218 | 0.019 | 0.02 | 1 | 0.841, p=0.098 | 0.297 | 0.245 | 0.09 |  |  |  |
| **Ankle extensors left** | Pre-treatment |  |  |  |  |  |  |  |  | 0.005 | 0.786 | 0.592 |
|  | Number of sessions |  |  |  |  |  |  |  |  | 0.005 | 0.786 | 0.592 |
|  | Months of hospital admission |  |  |  |  |  |  |  |  | 0.005 | 0.786 | 0.592 |
|  | Delay to start exercise program |  |  |  |  |  |  |  |  | 0.005 | 0.786 | 0.592 |
|  | Age |  |  |  |  |  |  |  |  | 0.005 | 0.786 | 0.592 |
| **Ankle extensors right** | Pre-treatment | 0.633 | 1 | 0.038 | 0.021 | 1.192, p=0.905 | 0.228 | <0.001 | 0.158 |  |  |  |
|  | Number of sessions | 0.33 | 0.279 | 1 | 0.071 | 1.108, p=0.792 | 0.228 | <0.001 | 0.158 |  |  |  |
|  | Months of hospital admission | 0.648 | 0.291 | 0.003 | 0.222 | 0.904, p=0.182 | 0.228 | <0.001 | 0.158 |  |  |  |
|  | Delay to start exercise program | 0.469 | 0.126 | 0.027 | 0.01 | 0.833, p=0.108 | 0.228 | <0.001 | 0.158 |  |  |  |
|  | Age | 0.414 | 0.006 | 0.003 | 1 | 1.161, p=0.83 | 0.228 | <0.001 | 0.158 |  |  |  |
| **Overall strength lower limbs left** | Pre-treatment | 0.63 | 1 | 0.169 | 0.057 | 1.141, p=0.828 | 0.22 | 0.193 | 0.562 |  |  |  |
|  | Number of sessions | 0.746 | 0.218 | 1 | 0.039 | 0.978, p=0.38 | 0.22 | 0.193 | 0.562 |  |  |  |
|  | Months of hospital admission | 0.67 | 0.209 | 0.457 | 0.082 | 1.049, p=0.608 | 0.22 | 0.193 | 0.562 |  |  |  |
|  | Delay to start exercise program | 0.34 | 0.142 | 0.029 | 0.015 | 0.911, p=0.23 | 0.22 | 0.193 | 0.562 |  |  |  |
|  | Age | 0.276 | 0.023 | 0.042 | 1 | 0.844, p=0.122 | 0.22 | 0.193 | 0.562 |  |  |  |
| **Overall strength lower limbs right** | Pre-treatment | 0.684 | 1 | 0.125 | 0.021 | 1.128, p=0.843 | 0.152 | 0.071 | 0.06 |  |  |  |
|  | Number of sessions | 0.452 | 0.291 | 1 | 0.053 | 1.094, p=0.743 | 0.152 | 0.071 | 0.06 |  |  |  |
|  | Months of hospital admission | 0.637 | 0.245 | 0.089 | 0.086 | 0.883, p=0.155 | 0.152 | 0.071 | 0.06 |  |  |  |
|  | Delay to start exercise program | 0.403 | 0.144 | 0.012 | 0.016 | 0.984, p=0.382 | 0.152 | 0.071 | 0.06 |  |  |  |
|  | Age | 0.232 | 0.015 | 0.006 | 1 | 0.949, p=0.305 | 0.152 | 0.071 | 0.06 |  |  |  |
| **6 minutes walking test/push test (mtrs)** | Pre-treatment | 0.614 | 1 | 0.105 | 0.05 | 1.053, p=0.59 | 0.299 | <0.001 | 0.898 |  |  |  |
|  | Number of sessions | 0.8 | 0.079 | 1 | 0.043 | 0.874, p=0.122 | 0.299 | <0.001 | 0.898 |  |  |  |
|  | Months of hospital admission | 0.624 | 0.196 | 0.449 | 0.104 | 1.251, p=0.958 | 0.299 | <0.001 | 0.898 |  |  |  |
|  | Delay to start exercise program | 0.322 | 0.013 | 0.023 | 0.02 | 1.161, p=0.863 | 0.299 | <0.001 | 0.898 |  |  |  |
|  | Age | 0.309 | 0.094 | 0.033 | 1 | 1.025, p=0.56 | 0.299 | <0.001 | 0.898 |  |  |  |
| **6 minutes walking test/push test asistance level** | Pre-treatment | 0.57 | 1 | 0.139 | 0.114 | 0.678, p=0.007 | 0.481 | 0.051 | 0.336 |  |  |  |
|  | Number of sessions | 0.736 | 0.19 | 1 | 0.039 | 0.914, p=0.198 | 0.481 | 0.051 | 0.336 |  |  |  |
|  | Months of hospital admission | 0.645 | 0.401 | 0.457 | 0.082 | 0.853, p=0.1 | 0.481 | 0.051 | 0.336 |  |  |  |
|  | Delay to start exercise program | 0.482 | 0.065 | 0.029 | 0.015 | 1.156, p=0.858 | 0.481 | 0.051 | 0.336 |  |  |  |
|  | Age | 0.331 | 0.085 | 0.042 | 1 | 0.824, p=0.05 | 0.481 | 0.051 | 0.336 |  |  |  |

*: Values of 1 corresponding to the concurvity of a variable with itself have been deleted to increase clarity and values higher than 0.8 are shown in red.

LM: Linear model; GAM: Generalized additive model.

^a^significant if p<0.05 (shown in red).

**Supplementary material. Figure 1:** Item response density plots.


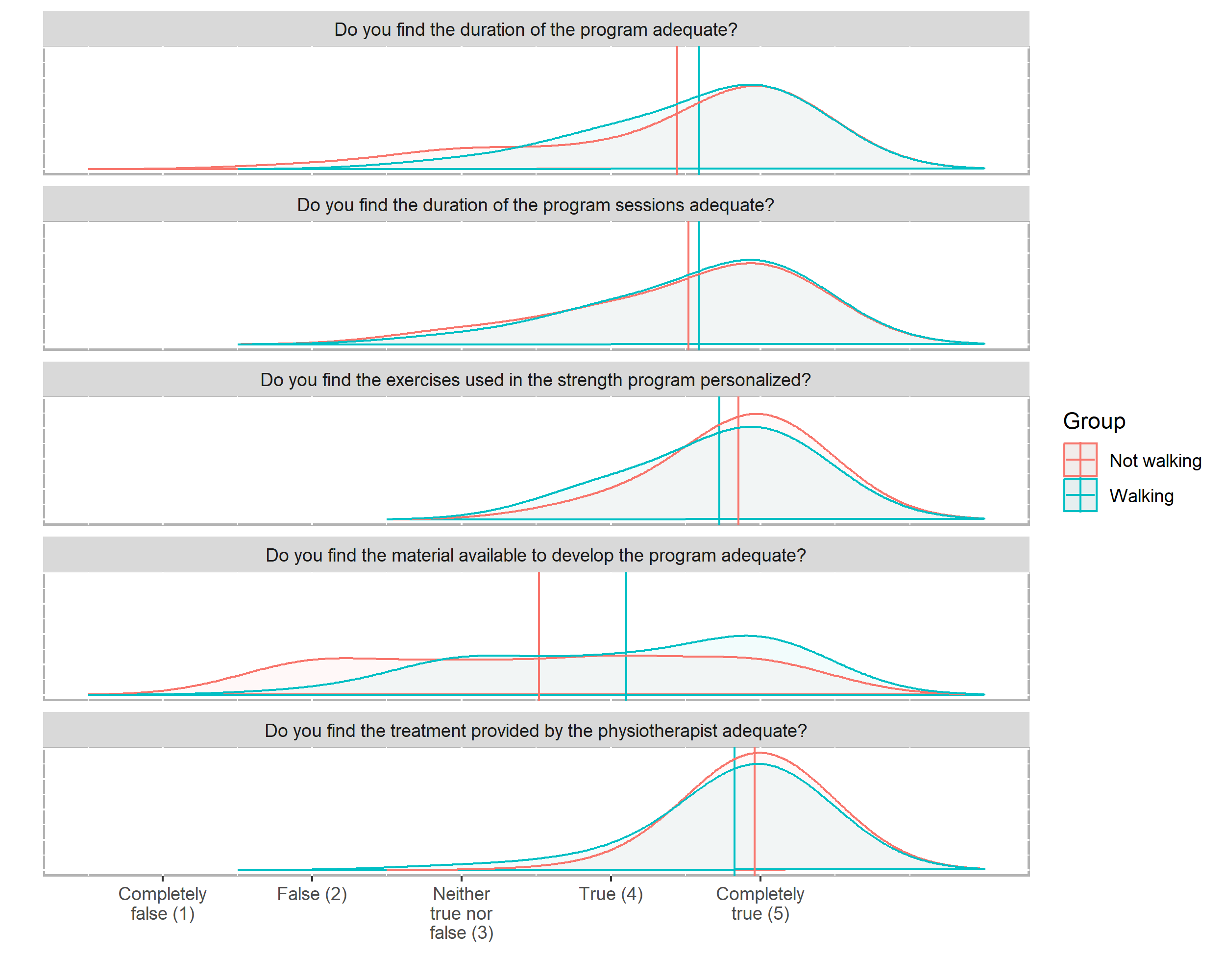


**Supplementary material. Figure 2:** Item response heatmap.


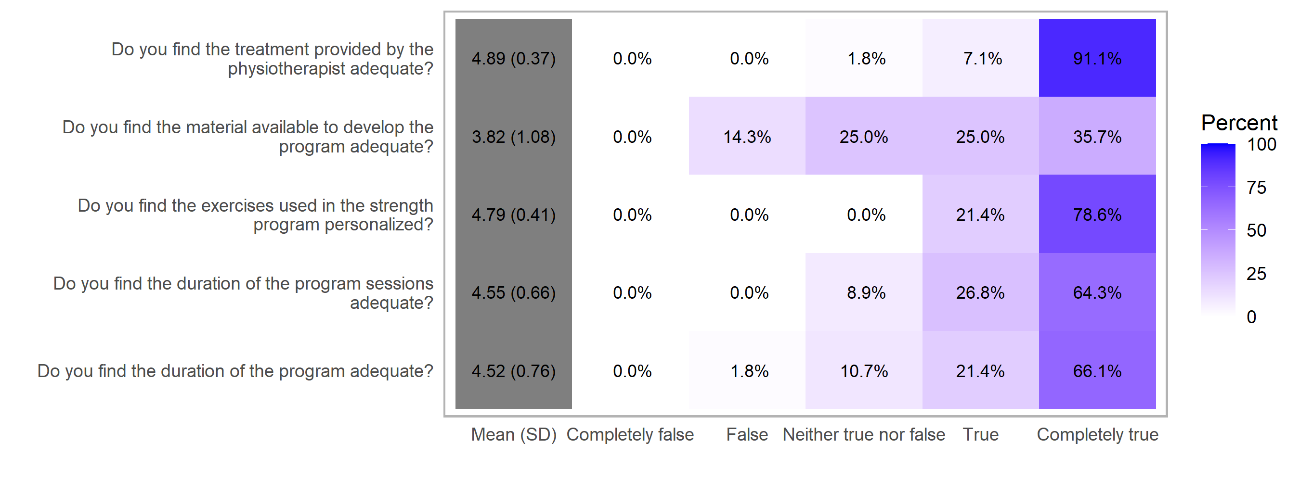

Supplement: Supplementary file 1 — Supplementary material 1 [file 12984_2025_1845_MOESM1_ESM.docx]
